# Enhanced Superconductivity in X_4_H_15_ Compounds via Hole‐Doping at Ambient Pressure

**DOI:** 10.1002/advs.202508419

**Published:** 2025-07-27

**Authors:** Kun Gao, Wenwen Cui, Tiago F. T. Cerqueira, Hai‐Chen Wang, Silvana Botti, Miguel A. L. Marques

**Affiliations:** ^1^ Research Center Future Energy Materials and Systems of the University Alliance Ruhr and Interdisciplinary Centre for Advanced Materials Simulation Ruhr University Bochum Universitätsstraße 150 D‐44801 Bochum Germany; ^2^ Laboratory of Quantum Materials Design and Application School of Physics and Electronic Engineering Jiangsu Normal University Xuzhou 221116 China; ^3^ CFisUC, Department of Physics University of Coimbra Rua Larga Coimbra 3004‐516 Portugal; ^4^ Faculty of Physics and Astronomy Ruhr University Bochum Universitätsstraße 150 D‐44801 Bochum Germany

**Keywords:** conventional superconductors, DFT calculations, hydride, hole‐doping, superconductivity

## Abstract

This study presents a computational investigation of X_4_H_15_ compounds (where X represents a metal) as potential superconductors at ambient conditions or under pressure. Through systematic density functional theory calculations and electron–phonon coupling analysis, it is demonstrated that electronic structure engineering via hole doping dramatically enhances the superconducting properties of these materials. While electron‐doped compounds with X^4 +^ cations (Ti, Zr, Hf, Th) exhibit modest transition temperatures of 1–9 K, hole‐doped systems with X^3 +^ cations (Y, Tb, Dy, Ho, Er, Tm, Lu) show remarkably higher values of ≈50 K at ambient pressure. Superconductivity in hole‐doped compounds originates from stronger coupling between electrons and both cation and hydrogen phonon modes. Although pristine X^3 +^_4_H_15_ compounds are thermodynamically unstable, a viable synthesis route via controlled hole doping of the charge‐compensated YZr_3_H_15_ compound is proposed. The calculations predict that even minimal concentrations of excess Y can induce high‐temperature superconductivity while preserving structural integrity. This work reveals how strategic electronic structure modulation can optimize superconducting properties in hydride systems, establishing a promising pathway toward practical high‐temperature conventional superconductors at ambient pressure.

## Graphical abstract

Hole doping in X_4_H_15_ hydrides significantly enhances superconductivity, with X^3 +^ compounds reaching *T*_c_ values near 50 K due to strong electron–phonon coupling. A viable route is proposed by doping thermodynamically unstable $X_{4}^{3+}H$_15_ to control the charge‐compensated YZr_3_H_15_ compound. This work demonstrates how electronic structure modulation can enable high‐*T*_c_ superconductivity under ambient pressure.

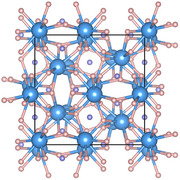

## 1 Introduction

Hydride superconductors have attracted significant attention due to their potential for achieving high‐temperature superconductivity, particularly in high‐pressure synthesized binary hydrides, such as H_3_S (203 K, 155 GPa)^[^ ^1^, ^2^, ^3^ ^]^ and LaH_10_ (250–260 K, 170 GPa),^[^ ^4^, ^5^, ^6^, ^7^ ^]^ and ternary hydrides, such as (La, Y)H_10_ (253 K at 183 GPa),^[^ ^8^ ^]^ (La, Ca)H_10_ (247 K at 173 GPa),^[^ ^9^ ^]^ and (La, Al)H_10_ (223 K at 164 GPa).^[^ ^10^ ^]^ These structures exhibit remarkably high superconducting transition temperature (*T*_c_), yet their stabilization pressures exceed 150 GPa, posing a significant barrier to practical applications. Consequently, researchers have shifted their focus toward identifying hydrides that maintain stability under relatively low‐pressure conditions or even at ambient temperatures, with the goal of discovering practical, novel superconductors.

Among these systems, the X_4_H_15_ compounds (where X = Zr, Hf, Th) represent a captivating class of materials, requiring relatively moderate pressures compared to other hydride superconductors. Th_4_H_15_, first synthesized and characterized in the 1970s, exhibits a transition temperature (*T*_c_) of 7.5–8 K at ambient pressure, establishing it as a pioneering example of hydride superconductivity.^[^ ^11^, ^12^ ^]^ The Th sublattice in Th_4_H_15_ adopts a cI16 structure, characterized as a distorted 2 × 2 × 2 supercell of a body‐centered cubic (bcc) sublattice.^[^ ^12^ ^]^ The hydrogen atoms in Th_4_H_15_ form an intricate network, incorporating both interstitial and framework hydrogen. This distinctive hydrogen arrangement, with multiple interstitial site occupations, profoundly influences the electronic and vibrational properties, thereby controlling superconductivity. Subsequently, Hf_4_H_15_ and Zr_4_H_15_ were successfully synthesized with analogous structures,^[^ ^13^, ^14^ ^]^ confirming the robustness and versatility of this hydride class. Experimental investigations reveal that Hf_4_H_15_ achieves a *T*_c_ of 4.5 K at 23 GPa,^[^ ^13^ ^]^ while Zr_4_H_15_ demonstrates superconductivity with a *T*_c_ of 4 K at 40 GPa.^[^ ^14^ ^]^ Computational studies have provided crucial insights into the stability and electronic properties of these systems. Theoretical calculations for Hf_4_H_15_ predict superconductivity with *T*_c_ ranging from 0.8 to 2.1 K at 200 GPa, maintaining structural stability between 100 and 200 GPa.^[^ ^15^ ^]^ Similarly, simulations for Zr_4_H_15_ predict a *T*_c_ of 0.2–0.8 K at 40 GPa, with stability in the 50–100 GPa range.^[^ ^14^ ^]^

The uniqueness of the X_4_H_15_ structure resides in its high hydrogen content and distinctive electronic properties, positioning it as an ideal candidate for investigating superconductivity in low‐pressure or ambient‐pressure hydrides. In this work, we present a comprehensive computational investigation of the X_4_H_15_ family, methodically exploring potential compounds where X spans the periodic table. Our study integrates thermodynamic stability analysis with electron‐phonon coupling calculations to predict superconducting properties. The objective is to elucidate the fundamental mechanisms driving superconductivity and develop optimization strategies for achieving higher *T*_c_. Our investigation reveals that hole doping emerges as a remarkably effective approach for enhancing superconductivity. By introducing hole carriers, selective doping strategically modulates the electronic density of states (DOS) near the Fermi level and significantly strengthens electron‐phonon coupling, offering a powerful new paradigm for optimizing the superconducting properties of hydrides.^[^ ^16^, ^17^, ^18^, ^19^ ^]^

We remark that previous theoretical studies have demonstrated that hole doping in hydrides such as CaYH_12_,^[^ ^20^ ^]^ Ca(BH_4_)_2_^[^ ^21^ ^]^ can strongly increase *T*_c_. This enhancement primarily stems from critical modifications in the DOS near the Fermi level and intensification of phonon softening effects induced by hole doping, which synergistically amplify electron–phonon coupling. Furthermore, hole doping not only potentially triggers new superconducting phases but also substantially reduces the pressure threshold required for achieving high‐temperature superconductivity, hence offering a viable pathway for exploring high‐*T*_c_ materials under ambient or near‐ambient conditions.^[^ ^22^, ^23^ ^]^ Experimentally, significant advancements have been made in realizing hole doping through elemental substitution or the introduction of vacancy defects. For instance, in the LaH_10_ system, partial substitution of La with low‐valence elements such as Be effectively introduces hole carriers, optimizing electron‐phonon coupling and enhancing superconducting performance.^[^ ^24^ ^]^ Additionally, hole doping in hydrogen storage materials like Mg(BH_4_)_2_ has successfully induced an insulator‐to‐metal transition, accompanied by an exceptionally high DOS near the Fermi level, resulting in a *T*_c_ as high as 140 K.^[^ ^25^ ^]^

## 2 Results and Discussion

**Figure 1:**
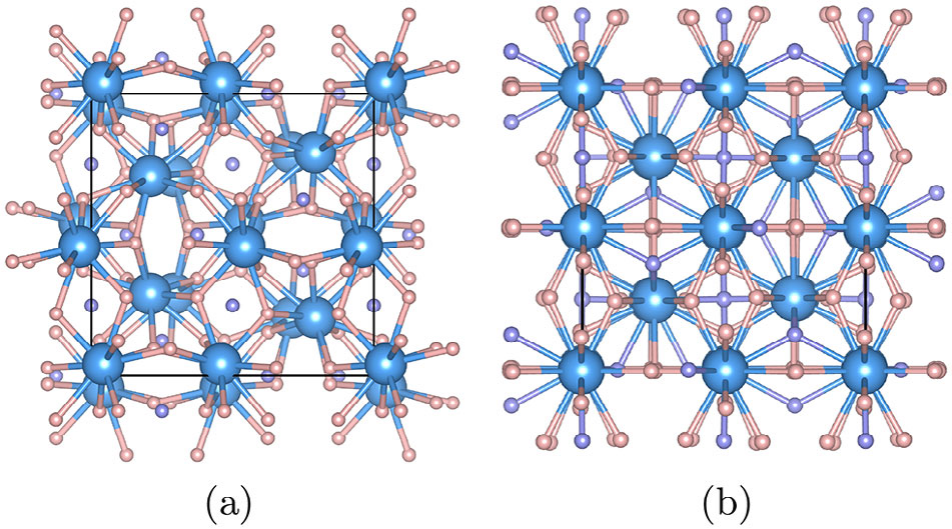
The structures of X_4_H_15_ with space group $I\bar{4}3d$ at a) 0 GPa and b) 80 GPa. Blue, pink, and purple spheres denote the X atoms at 16c, H atoms at 48e, and H atoms at 12a Wyckoff positions, respectively.

The compound X_4_H_15_ crystallizes in the cubic system with space group $I\bar{4}3d$ (#220) (see **Figure** **1**a). The X atoms exclusively occupy the 16*c* Wyckoff positions with full occupancy, while the hydrogen atoms distribute across two distinct crystallographic sites: the general 48*e* positions and the more symmetric 12*a* positions. This arrangement yields a total of 16 metal X atoms and 60 H atoms per conventional unit cell (4 formula units). The metal atoms in the conventional cell can be seen following a distorted bcc configuration. Each X atom is coordinated to 12 H atoms forming a distorted icosahedron. Neighboring icosahedra share facets formed by three H atoms. The strategic positioning of hydrogen atoms around the X centers creates well‐defined channels for electron transport and phonon propagation, playing a decisive role in determining the superconducting properties of these materials.

We can assume the ‐1 oxidation state of hydrogen, which is typical of hydrides, this assumption is supported by calculated Bader charges for Zr_4_H_15_ and Y_4_H_15_. The H atoms exhibit charges ranging from –0.45 to –0.49, while the cations Zr and Y carry charges of ≈+1.78 and +1.72, respectively. Although the charge transfer deviates from the idealized one‐electron transfer from cation to hydrogen, these values are typical for metal hydrides with hydrogen in the –1 oxidation state.^[^ ^26^, ^27^ ^]^

Achieving charge neutrality with the X_4_H^−1^15 stoichiometry proves impossible, as pristine X_4_H_15_ would necessitate an oxidation state of +3.75 for the X atom. The three experimentally confirmed systems incorporate X atoms with oxidation state +4, resulting in one excess electron per formula unit relative to charge compensation. Conversely, a +3 cation generates three holes, while other oxidation states produce an untenable quantity of electrons or holes that would likely destabilize the system, for example Sr_4_H_15_ and Ba_4_H_15_ (that have seven holes) deform considerably to a structure without any symmetry after geometry optimization. Consequently, it is logical that all X_4_H_15_ systems exhibiting semiconducting behavior in our calculations (B_4_H_15_, Cd_4_H_15_, N_4_H_15_, Tl_4_H_15_, Bi_4_H_15_, C_4_H_15_, O_4_H_15_, Si_4_H_15_, Cu_4_H_15_, Os_4_H_15_, Ru_4_H_15_, and La_4_H_15_) relax to crystal structures that deviate substantially from the prototypical Th_4_H_15_ arrangement.

A number of X_4_H_15_ are magnetic, which hinders conventional superconductivity, specifically the compounds with X = K, Mn, Tc, Cr, V, Ce, Re, Bi, Gd, U, Pu, Np. Of the remaining 35 compounds, that are metallic and non‐magnetic, only Zr_4_H_15_, Hf_4_H_15_, and Th_4_H_15_ are on the convex hull of stability. Note that these three compounds have been synthesized experimentally, in excellent agreement with our results. Up to 100 meV atom^−1^ of the convex hull we still find Np_4_H_15_ (26 meV atom^−1^), Ce_4_H_15_ (37 meV atom^−1^), Pu_4_H_15_ (81 meV atom^−1^), K_4_H_15_ (91 meV atom^−1^), and Ti_4_H_15_ (95 meV atom^−1^). We note that the convex hull in this work were calculated using the Alexandria database.^[^ ^28^, ^29^ ^]^

A possible way to improve the thermodynamic stability of this compounds is by enforcing charge compensation. To demonstrate this concept, we constructed YZr_3_H_15_ by substituting one Zr^4 +^ in Zr_4_H_15_ with Y^3 +^. This turns out to be a very stable compound, positioned merely 5 meV atom^−1^ from the convex hull of stability, even if Y_4_H_15_ is 233 meV atom^−1^ above the hull. While hull distances suggest YZr_3_H_15_ is less stable than Zr_4_H_15_ and Hf_4_H_15_, formation energies reveal the opposite: YZr_3_H_15_ (–0.466 eV atom^−1^) is more stable than Zr_4_H_15_ (–0.429 eV atom^−1^) or Y_4_H_15_ (–0.365 eV atom^−1^). This apparent contradiction arises because Y–H binaries are more stable than Zr–H binaries (see phase‐diagrams in the Supporting Information), increasing the distance to the hull of the ternary compound. Therefore, YZr_3_H_15_ is indeed more stable than Zr_4_H_15_ and Hf_4_H_15_, but this stability is offset by the higher stability of YH_3_.

**Figure 2:**
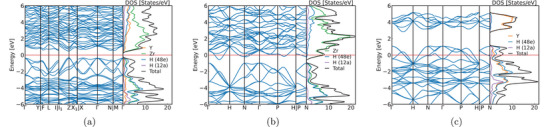
Electronic band structure and density of states (DOS) for representative compounds: a) charge‐compensated YZr_3_H_15_, b) electron‐doped Zr_4_H_15_, and c) hole‐doped Y_4_H_15_. The Fermi level is set at 0 eV.

As anticipated, this compound behaves as a semiconductor with an indirect band gap of ≈1.1 eV within the PBE approximation (see **Figure** **2**a). The valence band exhibits triple degeneracy at Γ, with one band displaying large hole mass along specific directions in the Brillouin zone. This characteristic generates a steep increase in the electronic density of states immediately below the valence band maximum. These bands predominantly comprise hydrogen states hybridized with the cation states. In contrast, the conduction bands manifest primarily Zr character, with minimal contributions from both Y and H. Based on these fundamental electronic structure characteristics, we can readily anticipate that hole doping would prove substantially more effective than electron doping in facilitating conventional superconductivity in these materials.

**Figure 3:**
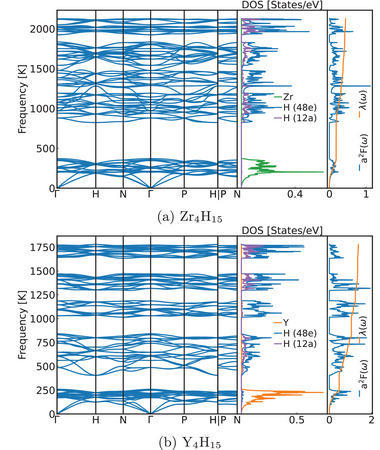
Phonon band structure, phonon density of states and Eliashberg spectral function α^2^ *F*(ω) for representative compounds: a) electron‐doped Zr_4_H_15_ and b) hole‐doped Y_4_H_15_. The stronger coupling with high‐frequency hydrogen modes in the hole‐doped compound is evident.

We begin our discussion on superconductivity with compounds where X = Ti, Zr, Hf, and Th exists in the +4 oxidation state. This configuration yields one excess electron per formula unit occupying the conduction band. The electronic band structure of Zr_4_H_15_, illustrated in Figure 2, exemplifies a degenerate semiconductor, exhibiting striking similarities to that of the semiconducting compound YZr_3_H_15_, presented in Figure 2a (note that the different symmetry points and path in the Brillouin zone result from the reduced symmetry of the latter system). The phonon band structure, depicted in **Figure** **3**a, demonstrates characteristics typical of hydrides with heavy cations, where acoustic and low‐lying optical modes consist exclusively of cation vibrations, while hydrogen governs the higher‐energy modes. The maximum phonon frequency exceeds 2000 K. Nevertheless, despite the Fermi energy residing at a pronounced peak in the density of states, this compound demonstrates an unexpectedly small electron–phonon coupling constant (λ), resulting in exceedingly modest superconducting transition temperatures (see **Table** **1**). Analogous behavior manifests in other X^4 +^ materials. The calculated *T*_c_ values for Zr and Hf compounds align closely with experimental measurements under pressure.^[^ ^13^, ^14^ ^]^ However, the predicted value for Th_4_H_15_ significantly underestimates the experimental observation (1.2 K vs the experimental 7–8 K.^[^ ^11^, ^12^ ^]^)

**Table 1:** Summary of the X_4_H_15_ compounds that are dynamically stable at zero pressure. We show the oxidation state of the X cation, the distance above the convex hull of stability (*E*_hull_ in eV atom^−1^), the superconducting transition temperature calculated with the Allen‐Dynes correction^[^ ^30^ ^]^ to the McMillan formula^[^ ^31^ ^]^ with μ* = 0.1 ($T_{c}^{\text{AD}}$ in K), the electron‐phonon coupling constant λ, average of electron‐phonon coupling matrix elements squared 〈*g* ^2^〉 (in eV^2^),^[^ ^32^ ^]^ the logarithmic average of the phonon frequency (ω_log_ in K), the total density of electronic states are Fermi level (TDOS in states eV^−1^ cell^−1^), and the partial density of H and X states ate the Fermi level (in states eV^−1^ cell^−1^).

Compound	oxi. state	*E*_hull_	$T_{c}^{\text{AD}}$	λ	〈*g* ^2^〉	ω_log_	TDOS	PDOS_H_	PDOS_X_
Y_4_H_15_	+3	0.233	53.1	1.32	0.89	474	5.38	3.45	1.87
Tb_4_H_15_	+3	0.232	48.6	1.30	1.58	440	5.14	3.28	1.83
Dy_4_H_15_	+3	0.231	48.9	1.27	1.61	457	5.05	3.20	1.81
Ho_4_H_15_	+3	0.230	48.9	1.24	1.63	475	4.97	3.14	1.80
Er_4_H_15_	+3	0.228	49.3	1.23	1.66	482	4.90	3.08	1.79
Tm_4_H_15_	+3	0.227	50.4	1.23	1.70	495	4.83	3.02	1.78
Lu_4_H_15_	+3	0.225	51.3	1.19	1.75	523	4.70	2.92	1.75
Th_4_H_15_	+4	0.000	1.2	0.38	0.36	368	15.24	0.46	14.40
Ti_4_H_15_	+4	0.095	9.2	0.52	0.13	625	9.82	0.49	9.15
Zr_4_H_15_	+4	0.000	2.9	0.41	0.25	607	7.84	0.51	7.16
Hf_4_H_15_	+4	0.000	4.8	0.47	0.57	476	7.33	0.53	6.65
Nb_4_H_15_	+5	0.129	34.4	1.28	0.40	319	9.49	0.63	8.64

In contrast, Nb_4_H_15_ containing Nb^5 +^, introduces five additional electrons into the conduction band. This substantial electron doping profoundly transforms the electronic structure, although the fundamental band architecture of the hypothetical charge‐compensated compound remains identifiable. The density of states at the Fermi level for this +5 compound is markedly elevated compared to its +4 counterparts, yielding electron‐phonon coupling constants exceeding unity (λ > 1) and correspondingly enhanced *T*_*c*_ values. Notably, the majority of coupling originates from the low‐lying niobium phonon modes, rather than from the high‐energy hydrogen vibrations, as demonstrated in SI #6 in Supporting Information.

The most intriguing results emerge for compounds containing trivalent metals: Y_4_H_15_, Tb_4_H_15_, Dy_4_H_15_, Ho_4_H_15_, Er_4_H_15_, Tm_4_H_15_, and Lu_4_H_15_. These systems, incorporating a +3 cation, generate three holes per formula unit in the valence band. All these compounds exhibit extraordinarily similar electronic and phononic band structures, culminating in nearly identical superconducting properties. The electronic band‐structure of Y_4_H_15_ is illustrated in Figure 2c. The resemblance to the charge‐compensated compound remains unmistakable, with the Fermi level positioned precisely on a prominent peak in the density of states. Once again, the low‐energy phonon states (see Figure 3) originate from the heavy cation, while the hydrogen modes segregate into distinct manifolds. The lowest energy and the two highest hydrogen manifolds involve vibrations of the atoms in both the 12a and 48e Wyckoff positions, while the remaining contains essentially vibrations of the hydrogens in the 48e positions. Due to the intricate geometry of these compounds, further categorizing these vibrations proves challenging; nevertheless, spectral analysis reveals that the low‐energy manifold predominantly exhibits torsional character, while the highest frequency modes correspond to hydrogen bond stretching vibrations. In this case, not only do the cation modes couple strongly to electrons, but the hydrogen modes contribute significantly as well, resulting in a substantial electron‐phonon coupling constant (λ = 1.3) and an exceptional superconducting transition temperature of ≈50 K (see Table 1).

The consistent behavior across diverse rare earth elements suggests that the superconducting properties are predominantly governed by common electronic structure features rather than the specific chemical identity of the metal. This fundamental observation receives further corroboration from our phonon calculations, which reveal strikingly similar phonon dispersions and electron–phonon coupling distributions across all trivalent metal compounds (see Figure 3 and Supporting Information).

Our comprehensive analysis spanning multiple compounds demonstrates that hole doping proves more effective than electron doping for enhancing superconducting properties in X_4_H_15_ systems. The λ values for hole‐doped compounds consistently exceed those of electron‐doped systems by a substantial margin, even when the total DOS at the Fermi level is comparatively lower (see Table 1). The profound contrast between hole‐doped and electron‐doped systems becomes evident, with the former exhibiting *T*_*c*_ values that surpass the latter by an order of magnitude. The electron‐phonon coupling parameter λ can also be expressed as λ = *N*(*E*_F_)〈*g* ^2^〉/*M*〈ω^2^〉,^[^ ^32^ ^]^ where *N*(*E*_F_), 〈*g* ^2^〉, 〈ω^2^〉, and *M* are the electronic density of states at the Fermi level, the average over the Fermi surface of electron‐phonon coupling matrix elements squared, the average of the phonon frequencies squared, and the atomic mass, respectively. As shown in Table 1, it happens that the hole doping $X_{4}^{3+}H$_15_ compounds have larger 〈*g* ^2^〉 values, therefore the increase of *T*_c_ should mainly be attributed to stronger electron‐phonon coupling instead of an increase of *N*(*E*_F_).

**Figure 4:**
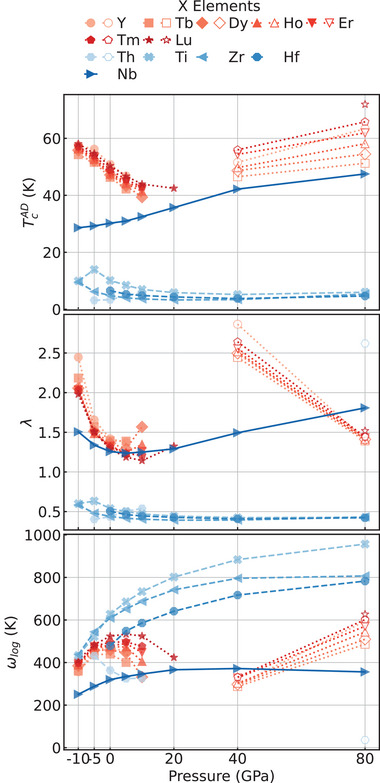
Superconducting critical temperature (*T*_c_), electron–phonon coupling constant (λ), and the logarithmic average of the phonon frequency (ω_log_) as a function of pressure. The filled and empty symbol represent the low‐ and high‐pressure structures (See Figure 1). For a certain pressure we omit the systems that are not dynamically stable. Note for Yb only PBE pseudopotential is available, for the sake of consistency we do not plot the curve of Yb_4_H_15_.

To understand how these materials behave under pressure, we performed calculations of the superconducting properties of X_4_H_15_ compounds with varying pressure from –10 to 80 GPa, (see **Figure** **4**). From the figure, the distinctive behavior of compounds depending on the oxidation state of the cation becomes immediately evident. For the +3 metals, λ decreases precipitously with pressure, while ω_log_ remains relatively constant, resulting in a monotonic decrease of *T*_c_. This phenomenon can be attributed to the softening of a phonon mode that eventually becomes imaginary at pressures above 10–20 GPa. The compounds subsequently undergo a structural phase transition (see Figure 1b) into a high‐pressure phase that maintains the same $I\bar{4}3d$ space group, but with cations reconfiguring into an undistorted bcc sublattice. In this structure, the electron‐phonon coupling decreases with pressure, while ω_log_ increases, leading to an enhancement of the transition temperature with pressure. For the +4 metals, λ diminishes to values substantially below 0.5, while ω_log_ increases to values approaching 1000 K, resulting in a net reduction of *T*_c_ with pressure. Finally, for Nb, λ exhibits a minimum at ≈5 GPa, while ω_log_ reaches a maximum at ≈40 GPa, yielding a monotonically increasing *T*_c_ that attains a value exceeding 40 K at 80 GPa.

We must emphasize that while $X_{4}^{3+}H$_15_ compounds demonstrate promising superconducting properties, they lack thermodynamic stability at ambient pressure. Our calculations reveal that these compounds reside ≈230 meV atom^−1^ above the convex hull of stability, and this instability can be directly attributed to the charge imbalance and the resulting electronic structure, which favors decomposition into more stable phases. As anticipated, the most probable decomposition pathway involves the charge‐compensated XH_3_ binary together with excess H_2_. While the formation enthalpy calculations indicate that these compounds become significantly more stable under pressure (see Supporting Information), a comprehensive assessment of their thermodynamic stability would require detailed phase diagram studies that are beyond the scope of this work.

While high‐pressure synthesis represents one possible route for these compounds, we propose a potentially more direct approach. Our strategy involves initiating with YZr_3_H_15_, which our calculations indicate lies remarkably close to thermodynamic stability (5 meV atom^−1^ above the convex hull) and exhibits chemical plausibility due to its charge‐compensated nature. Furthermore, cation site disorder will enhance stability through configurational entropy contributions. By synthesizing this compound with excess Y, one could effectively introduce hole doping while preserving structural integrity. As the DOS increases dramatically at the onset of valence states (see Figure 2), even modest amounts of excess Y might suffice to induce hole‐doped superconductivity in this system. Our band structure calculations demonstrate that removing merely 0.1–0.2 electrons per formula unit (equivalent to 5–10% Y excess) could shift the Fermi level into the region of elevated DOS at the top of the valence band.

To rigorously evaluate this concept, we constructed ordered cells with Y_2_Zr_2_H_15_ and Y_3_ZrH_15_ stoichiometry, for which the distance to the convex hull at 0 GPa are 59 and 135 meV atom^−1^, respectively. While these models cannot comprehensively capture the effects of substitutional doping in YZr_3_H_15_ due to the absence of disorder and the relatively high doping concentration, they provide critical insights into the potential behavior of such systems. The calculated electronic structure exhibits distinct metallic character with substantial DOS at the Fermi level, confirming the efficacy of this doping strategy. Furthermore, our phonon calculations for Y_2_Zr_2_H_15_ and Y_3_ZrH_15_ reveal no imaginary modes, unequivocally demonstrating dynamic stability. The electron‐phonon coupling constant for this compound is calculated to be ≈λ = 0.8 and 1.1. The *T*_c_ of these systems show an intermediate behavior with respect to the binary compounds, with *T*_c_ increasing with the amount of Y in the material. This intermediate behavior persists under applied pressure (as shown in Supporting Information).

In summary, our comprehensive computational investigation reveals that the superconducting properties of X_4_H_15_ compounds are profoundly influenced by their electronic configuration, with hole doping emerging as an exceptionally promising pathway to achieve high transition temperatures. The proposed strategy of introducing controlled hole doping in thermodynamically stable, charge‐compensated compounds represents a practical approach for the experimental realization of these promising superconducting materials.

Furthermore, our findings demonstrate that the underlying structural stability of these compounds is intimately connected to their electronic configuration. Charge‐compensated systems exhibit remarkable thermodynamic stability, while both electron and hole doping introduce varying degrees of instability that may necessitate high‐pressure synthesis conditions.

The extraordinary consistency in superconducting properties across different trivalent metal compounds (Y, Dy, Er, Ho, Lu, Tb, Tm) strongly indicates that the hole‐doping mechanism operates independently of the specific rare earth element. This universality reveals a fundamental electronic structure feature that could be systematically exploited in the rational design of new superconducting hydrides.

Our results carry significant implications for the broader field of hydride superconductivity. First, they underscore the critical importance of electronic structure engineering for optimizing superconducting properties. Second, they conclusively demonstrate that partial substitution constitutes an effective strategy for introducing carriers while maintaining structural stability. Finally, they suggest that other structurally related hydride systems could potentially benefit from analogous doping approaches.

## 3 Experimental Section

Calculations were performed of the entire family of X_4_H_15_ compounds where X spans the periodic table from Be to Bi, excluding rare‐gases. Geometry relaxations and total energy calculations were performed using the vasp code^[^ ^33^, ^34^ ^]^ with the Perdew–Burke–Ernzerhof approximation^[^ ^35^ ^]^ to the exchange‐correlation functional. To sample the Brillouin zones, a 3× 3 ×3 Γ‐centred k‐point grid was used. Spin‐polarised calculations were initiated from a ferromagnetic configuration. The projector augmented wave (PAW) setup^[^ ^36^, ^37^ ^]^ within vasp version 5.2 was utilized, applying a cutoff of 520 eV. We established the convergence criteria of the forces to be less than 0.005 eV Å^−1^.

Distances to the convex hull were calculated against the convex hull of the Alexandria database.^[^ ^28^, ^29^ ^]^ It is remarked that this represents the largest convex hull freely available, substantially more extensive than that of the Materials Project database.^[^ ^38^ ^]^ All parameters, including pseudopotentials, were configured to ensure compatibility with the data available in the Alexandria database.^[^ ^28^, ^29^ ^]^

Phonon calculations were executed using version 7.1 of quantum espresso ^[^ ^39^, ^40^ ^]^ with the Perdew–Burke–Ernzerhof functional for solids (PBEsol)^[^ ^35^ ^]^ generalized gradient approximation. The PBEsol pseudopotentials^[^ ^35^ ^]^ was employed from the pseudodojo project,^[^ ^41^ ^]^ specifically the stringent, scalar‐relativistic norm‐conserving set. Geometry optimizations were conducted using a uniform Γ‐centered 4 × 4 × 4 *k*‐point grid. Convergence thresholds for energies, forces, and stresses were established at 1 × 10^−8^ a.u., 1 × 10^−6^ a.u., and 5 × 10^−2^ kbar, respectively. For the electron–phonon coupling calculations, a double‐grid technique, utilizing a 8 × 8 × 8 *k*‐grid as the coarse grid, and a 16 × 16 × 16 as the fine grid was implemented. For the *q*‐sampling of phonons, a 2 × 2 × 2 *q*‐point grid was employed. The double δ‐integration to obtain the Eliashberg function was performed with a Methfessel–Paxton smearing of 0.05 Ry.

## Conflict of Interest

The authors declare no conflict of interest.

## Supporting information

Supporting Information

## Data Availability

The data that support the findings of this study are available in the supplementary material of this article.
