# Supplementary material for: Enhanced Superconductivity in X4H15 Compounds via Hole‐Doping at Ambient Pressure
Source: Adv Sci (Weinh). 2025 Jul 27;12(39):e08419. doi: 10.1002/advs.202508419 (PMC12533146; doi:10.1002/advs.202508419)
Supplement: Supplementary file 1 — Supporting Information [file ADVS-12-e08419-s001.pdf]

ADVANCED SCIENCE
Open Access

Supporting Information

for *Adv. Sci.*, DOI 10.1002/advs.202508419

Enhanced Superconductivity in X_4H_{15} Compounds via Hole-Doping at Ambient Pressure

Kun Gao, Wenwen Cui, Tiago F. T. Cerqueira, Hai-Chen Wang, Silvana Botti and Miguel A. L. Marques**

Supplementary Information for: Enhanced superconductivity in X_4H_{15} compounds via hole-doping at ambient pressure

Kun Gao,[1] Wenwen Cui,[2,*] Tiago F. T. Cerqueira,[3] Hai-Chen Wang,[1] Silvana Botti,[1] and Miguel A. L. Marques[1,†]

[1] *Research Center Future Energy Materials and Systems of the University Alliance Ruhr and Interdisciplinary Centre for Advanced Materials Simulation, Ruhr University Bochum, Universitätsstraße 150, D-44801 Bochum, Germany*
[2] *Laboratory of Quantum Materials Design and Application, School of Physics and Electronic Engineering, Jiangsu Normal University, Xuzhou 221116, China*
[3] *CFisUC, Department of Physics, University of Coimbra, Rua Larga, 3004-516 Coimbra, Portugal*

---

[*] wenwencui@jsnu.edu.cn
[†] miguel.marques@rub.de

#1: Dy_4H_{15}

**mat id**	agm006249793
**spg**	220
**nsites**	38
**e above hull**	0.231 eV
**e form**	-0.369 eV
**decomposition**	DyH_3, H_2
**ecutwfc**	84.0 Ry
**kpts coarse**	8×8×8
**kpts fine**	16×16×16
**qpts**	2×2×2
λ	1.272
$\omega_{\mathbf{log}}$	457 K
ω_2	778 K
$T_{\mathbf{c}}^{\mathbf{Mcmillan}}$	43.8 K
$T_{\mathbf{c}}^{\mathbf{Allen\text{-}Dynes}}$	48.9 K
$T_{\mathbf{c}}^{\mathbf{Eliashberg}}$	54.6 K

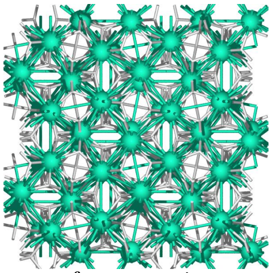

from a-axis

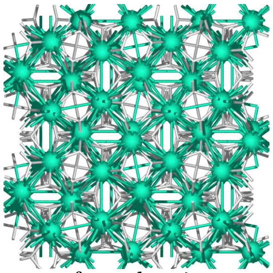

from b-axis

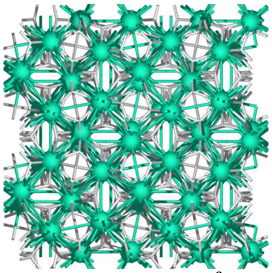

from c-axis

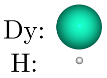

comment:
Accurate calculation.

Primitive structure:

a: 7.4657 Å, b: 7.4657 Å, c: 7.4657 Å
α: 109.47°, β: 109.47°, γ: 109.47°

Dy	[0.9169, 0.9169, 0.9169]
Dy	[0.0831, 0.5000, 1.0000]
Dy	[0.5000, 1.0000, 0.0831]
Dy	[0.0000, 0.0831, 0.5000]
Dy	[0.5000, 0.0000, 0.5831]
Dy	[0.0000, 0.5831, 0.5000]
Dy	[0.5831, 0.5000, 0.0000]
Dy	[0.4169, 0.4169, 0.4169]
H	[0.3741, 0.6553, 0.9712]
H	[0.8447, 0.2189, 0.3160]
H	[0.5288, 0.1840, 0.4029]
H	[0.1259, 0.0971, 0.2811]
H	[0.0971, 0.2811, 0.1259]
H	[0.2811, 0.1259, 0.0971]
H	[0.8741, 0.4712, 0.1553]
H	[0.8160, 0.7189, 0.3447]
H	[0.2500, 0.6250, 0.3750]
H	[0.3750, 0.2500, 0.6250]
H	[0.1250, 0.7500, 0.8750]
H	[0.8750, 0.1250, 0.7500]
H	[0.6250, 0.3750, 0.2500]
H	[0.7500, 0.8750, 0.1250]
H	[0.6259, 0.7811, 0.5971]
H	[0.2189, 0.3160, 0.8447]
H	[0.9712, 0.3741, 0.6553]
H	[0.6553, 0.9712, 0.3741]
H	[0.4029, 0.5288, 0.1840]
H	[0.1840, 0.4029, 0.5288]
H	[0.3160, 0.8447, 0.2189]
H	[0.0288, 0.9029, 0.6840]
H	[0.3447, 0.8160, 0.7189]
H	[0.4712, 0.1553, 0.8741]
H	[0.9029, 0.6840, 0.0288]
H	[0.7189, 0.3447, 0.8160]
H	[0.1553, 0.8741, 0.4712]
H	[0.5971, 0.6259, 0.7811]
H	[0.7811, 0.5971, 0.6259]
H	[0.6840, 0.0288, 0.9029]

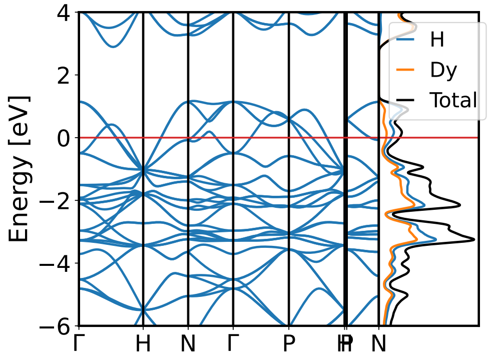

Electron band structure

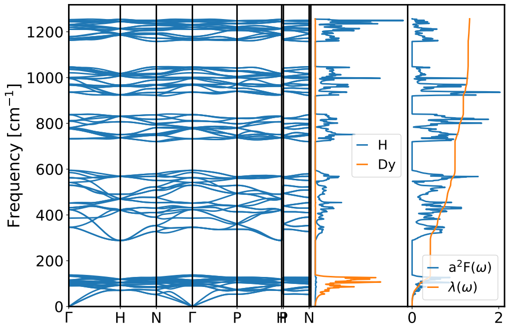

Phonon band structure

#2: Er_4H_{15}

**mat id**	agm006249797
**spg**	220
**nsites**	38
**e above hull**	0.228 eV
**e form**	-0.375 eV
**decomposition**	ErH_3, H_2
**ecutwfc**	84.0 Ry
**kpts coarse**	8×8×8
**kpts fine**	16×16×16
**qpts**	2×2×2
λ	1.230
$\omega_{\log}$	482 K
ω_2	811 K
T_c^{Mcmillan}	44.5 K
$T_c^{\mathrm{Allen\text{-}Dynes}}$	49.3 K
$T_c^{\mathrm{Eliashberg}}$	55.1 K

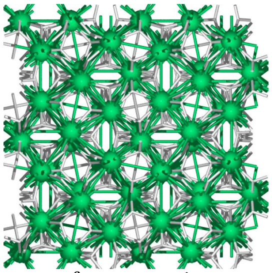

from a-axis

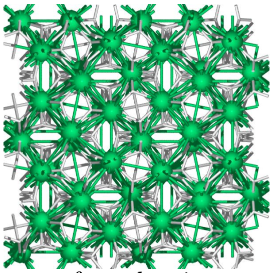

from b-axis

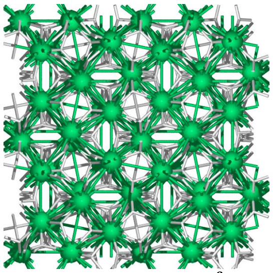

from c-axis

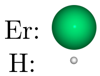

comment:
Accurate calculation.

Primitive structure:

a: 7.3785 Å, b: 7.3785 Å, c: 7.3785 Å
α: 109.47°, β: 109.47°, γ: 109.47°

Er [0.0000, 0.9145, 0.5000]
Er [0.0855, 0.0855, 0.0855]
Er [0.4145, 0.5000, 0.0000]
Er [0.5000, 0.0000, 0.4145]
Er [0.9145, 0.5000, 0.0000]
Er [0.5000, 1.0000, 0.9145]
Er [0.5855, 0.5855, 0.5855]
Er [0.0000, 0.4145, 0.5000]
H [0.4047, 0.3778, 0.2212]
H [0.5269, 0.8435, 0.1222]
H [0.1222, 0.5269, 0.8435]
H [0.8435, 0.1222, 0.5269]
H [0.9731, 0.0953, 0.3165]
H [0.1835, 0.2788, 0.6565]
H [0.7212, 0.8778, 0.9047]
H [0.4731, 0.8165, 0.5953]
H [0.8750, 0.2500, 0.1250]
H [0.7500, 0.3750, 0.6250]
H [0.2500, 0.1250, 0.8750]
H [0.1250, 0.8750, 0.2500]
H [0.3750, 0.6250, 0.7500]
H [0.6250, 0.7500, 0.3750]
H [0.0269, 0.6222, 0.3435]
H [0.3778, 0.2212, 0.4047]
H [0.3165, 0.9731, 0.0953]
H [0.2788, 0.6565, 0.1835]
H [0.2212, 0.4047, 0.3778]
H [0.6565, 0.1835, 0.2788]
H [0.0953, 0.3165, 0.9731]
H [0.3435, 0.0269, 0.6222]
H [0.6222, 0.3435, 0.0269]
H [0.5953, 0.4731, 0.8165]
H [0.8778, 0.9047, 0.7212]
H [0.9047, 0.7212, 0.8778]
H [0.6835, 0.1565, 0.7788]
H [0.8165, 0.5953, 0.4731]
H [0.1565, 0.7788, 0.6835]
H [0.7788, 0.6835, 0.1565]

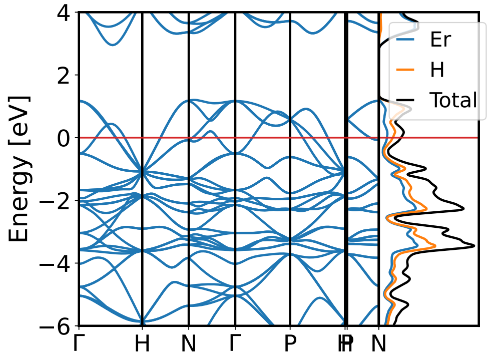

Electron band structure

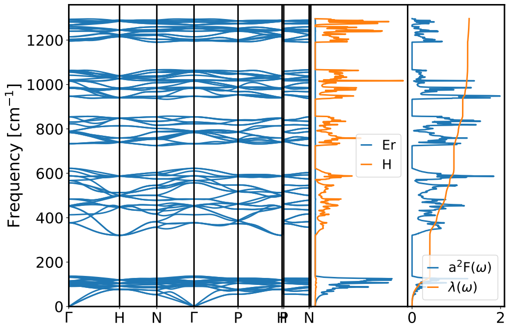

Phonon band structure

#3: Hf_4H_{15}

**mat id**	agm006249802
**spg**	220
**nsites**	38
**e above hull**	0.000 eV
**e form**	-0.418 eV
**decomposition**	Hf_4H_{15}
**ecutwfc**	144.0 Ry
**kpts coarse**	8×8×8
**kpts fine**	16×16×16
**qpts**	2×2×2
λ	0.474
$\omega_{\mathbf{log}}$	476 K
ω_2	1022 K
$T_{\mathbf{c}}^{\mathbf{Mcmillan}}$	4.7 K
$T_{\mathbf{c}}^{\mathbf{Allen\text{-}Dynes}}$	4.8 K
$T_{\mathbf{c}}^{\mathbf{Eliashberg}}$	4.3 K

comment:
Accurate calculation.

Primitive structure:

a: 6.8713 Å, b: 6.8713 Å, c: 6.8713 Å
α: 109.47°, β: 109.47°, γ: 109.47°

```
Hf  [0.9043, 0.9043, 0.9043]
Hf  [0.0957, 0.5000, 0.0000]
Hf  [0.5000, 0.0000, 0.0957]
Hf  [0.0000, 0.0957, 0.5000]
Hf  [0.5000, 0.0000, 0.5957]
Hf  [1.0000, 0.5957, 0.5000]
Hf  [0.5957, 0.5000, 1.0000]
Hf  [0.4043, 0.4043, 0.4043]
 H  [0.3866, 0.6551, 0.9667]
 H  [0.8449, 0.2315, 0.3116]
 H  [0.5333, 0.1884, 0.4199]
 H  [0.1134, 0.0801, 0.2685]
 H  [0.0801, 0.2685, 0.1134]
 H  [0.2685, 0.1134, 0.0801]
 H  [0.8866, 0.4667, 0.1551]
 H  [0.8116, 0.7315, 0.3449]
 H  [0.2500, 0.6250, 0.3750]
 H  [0.3750, 0.2500, 0.6250]
 H  [0.1250, 0.7500, 0.8750]
 H  [0.8750, 0.1250, 0.7500]
 H  [0.6250, 0.3750, 0.2500]
 H  [0.7500, 0.8750, 0.1250]
 H  [0.6134, 0.7685, 0.5801]
 H  [0.2315, 0.3116, 0.8449]
 H  [0.9667, 0.3866, 0.6551]
 H  [0.6551, 0.9667, 0.3866]
 H  [0.4199, 0.5333, 0.1884]
 H  [0.1884, 0.4199, 0.5333]
 H  [0.3116, 0.8449, 0.2315]
 H  [0.0333, 0.9199, 0.6884]
 H  [0.3449, 0.8116, 0.7315]
 H  [0.4667, 0.1551, 0.8866]
 H  [0.9199, 0.6884, 0.0333]
 H  [0.7315, 0.3449, 0.8116]
 H  [0.1551, 0.8866, 0.4667]
 H  [0.5801, 0.6134, 0.7685]
 H  [0.7685, 0.5801, 0.6134]
 H  [0.6884, 0.0333, 0.9199]
```

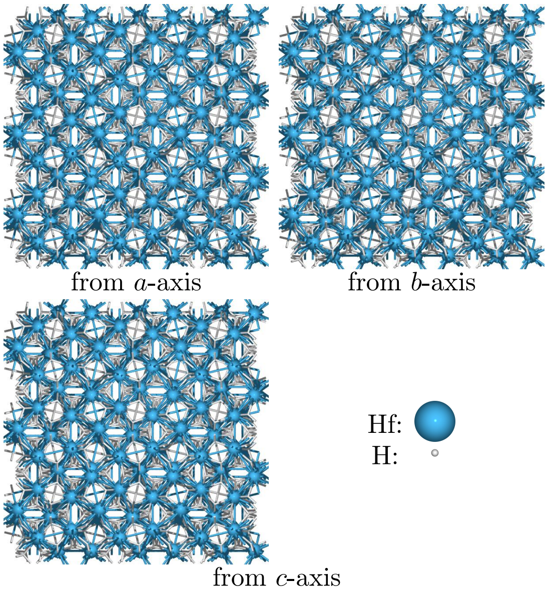

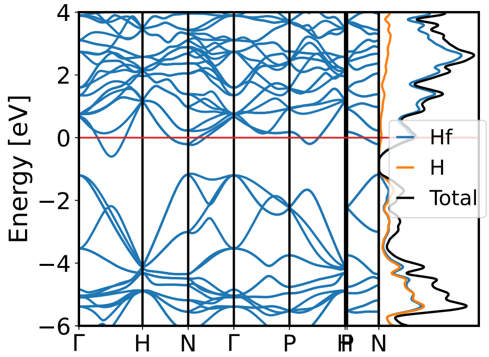

Electron band structure

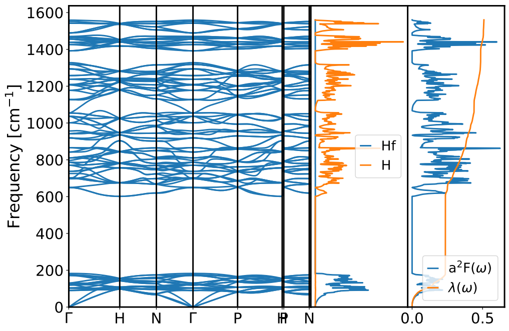

Phonon band structure

#4: Ho_4H_{15}

**mat id**	agm006249795
**spg**	220
**nsites**	38
**e above hull**	0.230 eV
**e form**	-0.372 eV
**decomposition**	HoH_3, H_2
**ecutwfc**	84.0 Ry
**kpts coarse**	8×8×8
**kpts fine**	16×16×16
**qpts**	2×2×2
λ	1.235
$\omega_{\mathbf{log}}$	475 K
ω_2	800 K
$T_{\mathbf{c}}^{\mathbf{Mcmillan}}$	44.0 K
$T_{\mathbf{c}}^{\mathbf{Allen\text{-}Dynes}}$	48.9 K
$T_{\mathbf{c}}^{\mathbf{Eliashberg}}$	54.5 K

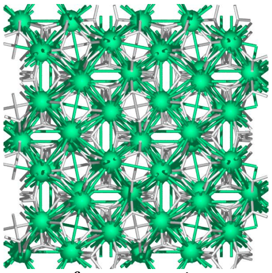

from *a*-axis

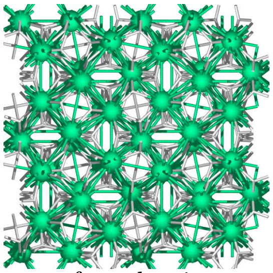

from *b*-axis

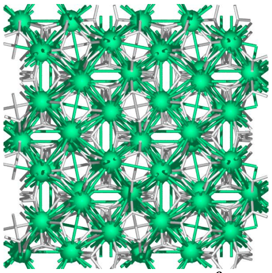

from *c*-axis

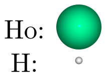

comment:
Accurate calculation.

Primitive structure:

a: 7.4221 Å, b: 7.4221 Å, c: 7.4221 Å
α: 109.47°, β: 109.47°, γ: 109.47°

Ho [1.0000, 0.9157, 0.5000]
Ho [0.0843, 0.0843, 0.0843]
Ho [0.4157, 0.5000, 0.0000]
Ho [0.5000, 1.0000, 0.4157]
Ho [0.9157, 0.5000, 1.0000]
Ho [0.5000, 1.0000, 0.9157]
Ho [0.5843, 0.5843, 0.5843]
Ho [0.0000, 0.4157, 0.5000]
H [0.4038, 0.3761, 0.2200]
H [0.5277, 0.8439, 0.1239]
H [0.1239, 0.5277, 0.8439]
H [0.8439, 0.1239, 0.5277]
H [0.9723, 0.0962, 0.3163]
H [0.1837, 0.2800, 0.6561]
H [0.7200, 0.8761, 0.9038]
H [0.4723, 0.8163, 0.5962]
H [0.8750, 0.2500, 0.1250]
H [0.7500, 0.3750, 0.6250]
H [0.2500, 0.1250, 0.8750]
H [0.1250, 0.8750, 0.2500]
H [0.3750, 0.6250, 0.7500]
H [0.6250, 0.7500, 0.3750]
H [0.0277, 0.6239, 0.3439]
H [0.3761, 0.2200, 0.4038]
H [0.3163, 0.9723, 0.0962]
H [0.2800, 0.6561, 0.1837]
H [0.2200, 0.4038, 0.3761]
H [0.6561, 0.1837, 0.2800]
H [0.0962, 0.3163, 0.9723]
H [0.3439, 0.0277, 0.6239]
H [0.6239, 0.3439, 0.0277]
H [0.5962, 0.4723, 0.8163]
H [0.8761, 0.9038, 0.7200]
H [0.9038, 0.7200, 0.8761]
H [0.6837, 0.1561, 0.7800]
H [0.8163, 0.5962, 0.4723]
H [0.1561, 0.7800, 0.6837]
H [0.7800, 0.6837, 0.1561]

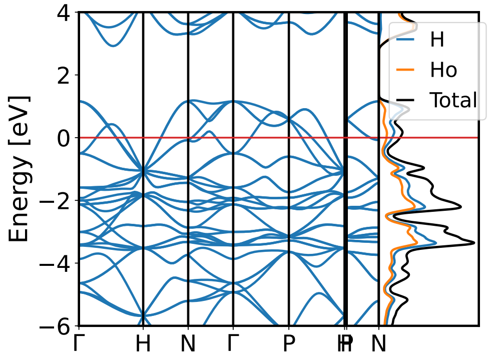

Electron band structure

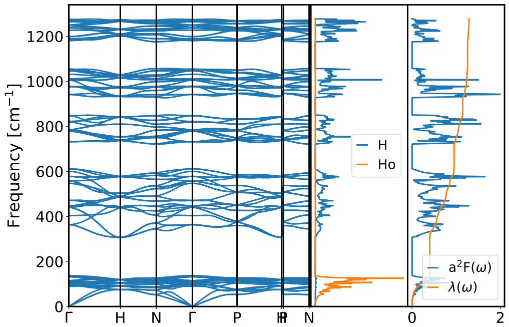

Phonon band structure

#5: Lu_4H_{15}

**mat id**	agm073026977
**spg**	220
**nsites**	38
**e above hull**	0.225 eV
**e form**	-0.370 eV
**decomposition**	LuH_3, H_2
**ecutwfc**	116.0 Ry
**kpts coarse**	8×8×8
**kpts fine**	16×16×16
**qpts**	2×2×2
λ	1.193
$\omega_{\mathbf{log}}$	523 K
ω_2	855 K
$T_{\mathbf{c}}^{\mathbf{Mcmillan}}$	46.5 K
$T_{\mathbf{c}}^{\mathbf{Allen\text{-}Dynes}}$	51.3 K
$T_{\mathbf{c}}^{\mathbf{Eliashberg}}$	57.3 K

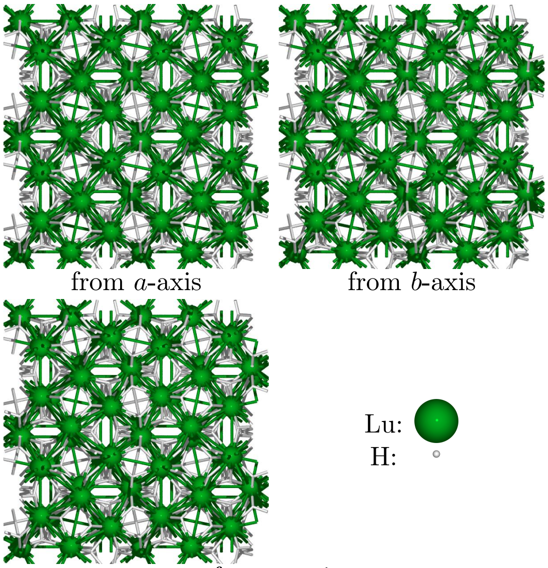

comment:
Accurate calculation.

Primitive structure:
a: 7.2487 Å, b: 7.2487 Å, c: 7.2487 Å
α: 109.47°, β: 109.47°, γ: 109.47°

Lu [0.5873, 0.5873, 0.5873]
Lu [0.5000, 0.0000, 0.9127]
Lu [0.0873, 0.0873, 0.0873]
Lu [0.4127, 0.5000, 1.0000]
Lu [0.9127, 0.5000, 0.0000]
Lu [0.5000, 1.0000, 0.4127]
Lu [1.0000, 0.4127, 0.5000]
Lu [1.0000, 0.9127, 0.5000]
H [0.9067, 0.7229, 0.8801]
H [0.1250, 0.8750, 0.2500]
H [0.4734, 0.8161, 0.5933]
H [0.8161, 0.5933, 0.4734]
H [0.7771, 0.6839, 0.1572]
H [0.8428, 0.1199, 0.5266]
H [0.3428, 0.0266, 0.6199]
H [0.9734, 0.0933, 0.3161]
H [0.1199, 0.5266, 0.8428]
H [0.0266, 0.6199, 0.3428]
H [0.8801, 0.9067, 0.7229]
H [0.2229, 0.4067, 0.3801]
H [0.8750, 0.2500, 0.1250]
H [0.7229, 0.8801, 0.9067]
H [0.6250, 0.7500, 0.3750]
H [0.2771, 0.6572, 0.1839]
H [0.6572, 0.1839, 0.2771]
H [0.1839, 0.2771, 0.6572]
H [0.3801, 0.2229, 0.4067]
H [0.2500, 0.1250, 0.8750]
H [0.6839, 0.1572, 0.7771]
H [0.1572, 0.7771, 0.6839]
H [0.3161, 0.9734, 0.0933]
H [0.6199, 0.3428, 0.0266]
H [0.4067, 0.3801, 0.2229]
H [0.5933, 0.4734, 0.8161]
H [0.5266, 0.8428, 0.1199]
H [0.3750, 0.6250, 0.7500]
H [0.0933, 0.3161, 0.9734]
H [0.7500, 0.3750, 0.6250]

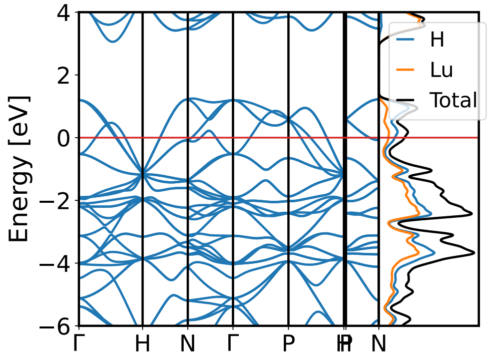

Electron band structure

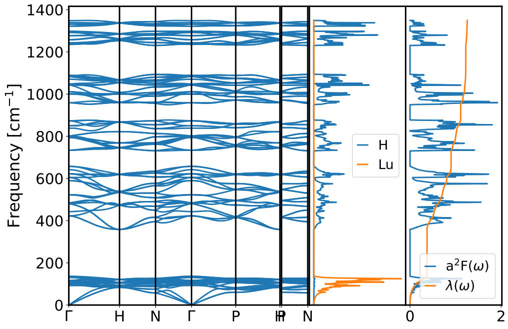

Phonon band structure

#6: Nb_4H_{15}

**mat id**	agm073024595
**spg**	220
**nsites**	38
**e above hull**	0.129 eV
**e form**	-0.081 eV
**decomposition**	NbH_2, H_2
**ecutwfc**	98.0 Ry
**kpts coarse**	8×8×8
**kpts fine**	16×16×16
**qpts**	2×2×2
λ	1.277
$\omega_{\mathbf{log}}$	319 K
ω_2	741 K
$T_{\mathbf{c}}^{\mathbf{Mcmillan}}$	30.8 K
$T_{\mathbf{c}}^{\mathbf{Allen\text{-}Dynes}}$	34.4 K
$T_{\mathbf{c}}^{\mathbf{Eliashberg}}$	34.3 K

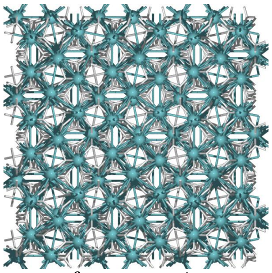

from a-axis

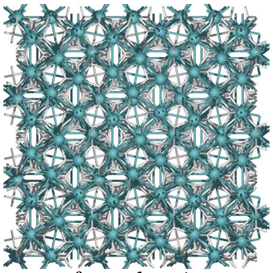

from b-axis

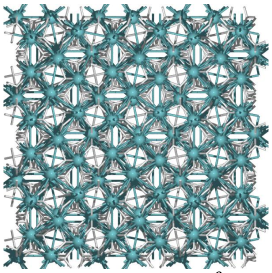

from c-axis

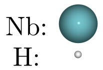

comment:
Accurate calculation.

Primitive structure:

a: 6.7138 Å, b: 6.7138 Å, c: 6.7138 Å
α: 109.47°, β: 109.47°, γ: 109.47°

Nb [0.5000, 1.0000, 0.9153]
Nb [0.0000, 0.9153, 0.5000]
Nb [0.5000, 0.0000, 0.4153]
Nb [0.4153, 0.5000, 0.0000]
Nb [0.9153, 0.5000, 0.0000]
Nb [1.0000, 0.4153, 0.5000]
Nb [0.0847, 0.0847, 0.0847]
Nb [0.5847, 0.5847, 0.5847]
H [0.6866, 0.1561, 0.7592]
H [0.7500, 0.3750, 0.6250]
H [0.1561, 0.7592, 0.6866]
H [0.7408, 0.8969, 0.9273]
H [0.5727, 0.4695, 0.8134]
H [0.2408, 0.4273, 0.3969]
H [0.8134, 0.5727, 0.4695]
H [0.3969, 0.2408, 0.4273]
H [0.0727, 0.3134, 0.9695]
H [0.9273, 0.7408, 0.8969]
H [0.4695, 0.8134, 0.5727]
H [0.3134, 0.9695, 0.0727]
H [0.1250, 0.8750, 0.2500]
H [0.3439, 0.0305, 0.6031]
H [0.3750, 0.6250, 0.7500]
H [0.1031, 0.5305, 0.8439]
H [0.9695, 0.0727, 0.3134]
H [0.4273, 0.3969, 0.2408]
H [0.6561, 0.1866, 0.2592]
H [0.6250, 0.7500, 0.3750]
H [0.0305, 0.6031, 0.3439]
H [0.8969, 0.9273, 0.7408]
H [0.8439, 0.1031, 0.5305]
H [0.7592, 0.6866, 0.1561]
H [0.5305, 0.8439, 0.1031]
H [0.6031, 0.3439, 0.0305]
H [0.1866, 0.2592, 0.6561]
H [0.2500, 0.1250, 0.8750]
H [0.2592, 0.6561, 0.1866]
H [0.8750, 0.2500, 0.1250]

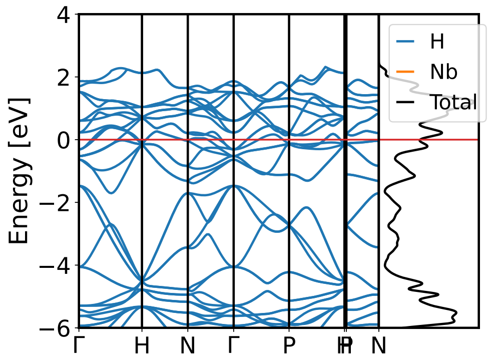

Electron band structure

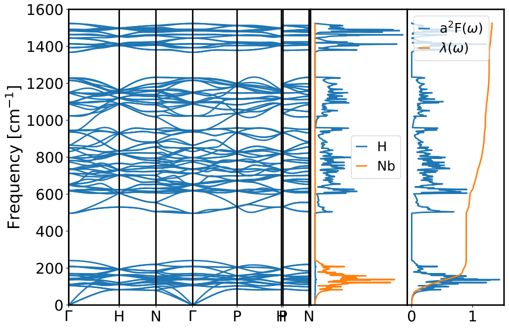

Phonon band structure

#7: Ta_4H_{15}

**mat id**	agm073024999
**spg**	220
**nsites**	38
**e above hull**	0.113 eV
**e form**	-0.014 eV
**decomposition**	TaH_2, H_2
**ecutwfc**	144.0 Ry
**kpts coarse**	8×8×8
**kpts fine**	16×16×16
**qpts**	2×2×2
λ	2.732
$\omega_{\mathbf{log}}$	116 K
ω_2	552 K
$T_{\mathbf{c}}^{\mathbf{Mcmillan}}$	20.0 K
$T_{\mathbf{c}}^{\mathbf{Allen\text{-}Dynes}}$	27.3 K
$T_{\mathbf{c}}^{\mathbf{Eliashberg}}$	38.4 K

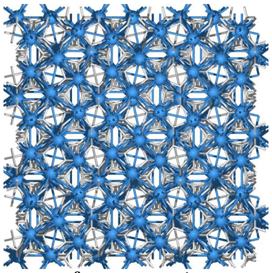

from a-axis

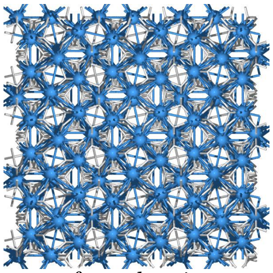

from b-axis

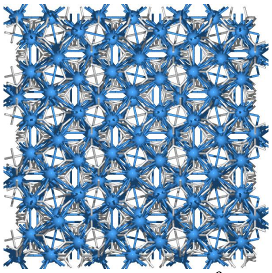

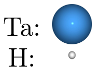

from c-axis

comment:
Accurate calculation.

Primitive structure:

a: 6.6741 Å, b: 6.6741 Å, c: 6.6741 Å
α: 109.47°, β: 109.47°, γ: 109.47°

```
Ta  [0.5000, 0.0000, 0.9092]
Ta  [0.0000, 0.9092, 0.5000]
Ta  [0.5000, 0.0000, 0.4092]
Ta  [0.4092, 0.5000, 0.0000]
Ta  [0.9092, 0.5000, 0.0000]
Ta  [1.0000, 0.4092, 0.5000]
Ta  [0.0908, 0.0908, 0.0908]
Ta  [0.5908, 0.5908, 0.5908]
H   [0.6875, 0.1562, 0.7593]
H   [0.7500, 0.3750, 0.6250]
H   [0.1562, 0.7593, 0.6875]
H   [0.7407, 0.8969, 0.9281]
H   [0.5719, 0.4687, 0.8125]
H   [0.2407, 0.4281, 0.3969]
H   [0.8125, 0.5719, 0.4687]
H   [0.3969, 0.2407, 0.4281]
H   [0.0719, 0.3125, 0.9687]
H   [0.9281, 0.7407, 0.8969]
H   [0.4687, 0.8125, 0.5719]
H   [0.3125, 0.9687, 0.0719]
H   [0.1250, 0.8750, 0.2500]
H   [0.3438, 0.0313, 0.6031]
H   [0.3750, 0.6250, 0.7500]
H   [0.1031, 0.5313, 0.8438]
H   [0.9687, 0.0719, 0.3125]
H   [0.4281, 0.3969, 0.2407]
H   [0.6562, 0.1875, 0.2593]
H   [0.6250, 0.7500, 0.3750]
H   [0.0313, 0.6031, 0.3438]
H   [0.8969, 0.9281, 0.7407]
H   [0.8438, 0.1031, 0.5313]
H   [0.7593, 0.6875, 0.1562]
H   [0.5313, 0.8438, 0.1031]
H   [0.6031, 0.3438, 0.0313]
H   [0.1875, 0.2593, 0.6562]
H   [0.2500, 0.1250, 0.8750]
H   [0.2593, 0.6562, 0.1875]
H   [0.8750, 0.2500, 0.1250]
```

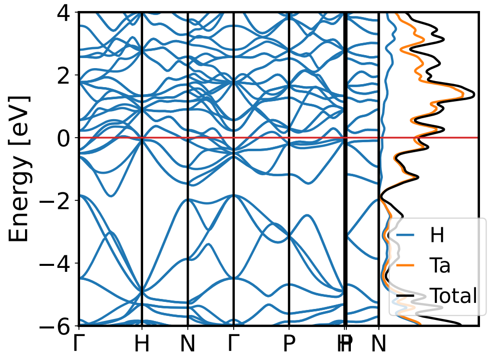

Electron band structure

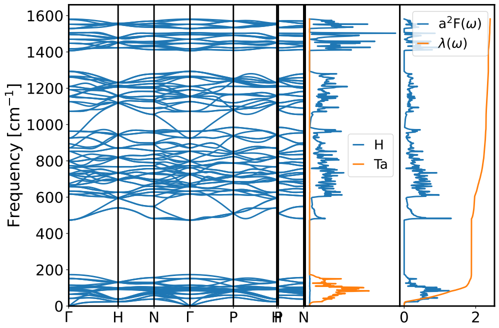

Phonon band structure

#8: Tb_4H_{15}

**mat id**	agm006249791
**spg**	220
**nsites**	38
**e above hull**	0.232 eV
**e form**	-0.364 eV
**decomposition**	TbH_3, H_2
**ecutwfc**	84.0 Ry
**kpts coarse**	8×8×8
**kpts fine**	16×16×16
**qpts**	2×2×2
λ	1.302
$\omega_{\mathbf{log}}$	440 K
ω_2	758 K
$T_{\mathbf{c}}^{\mathbf{Mcmillan}}$	43.4 K
$T_{\mathbf{c}}^{\mathbf{Allen\text{-}Dynes}}$	48.6 K
$T_{\mathbf{c}}^{\mathbf{Eliashberg}}$	54.3 K

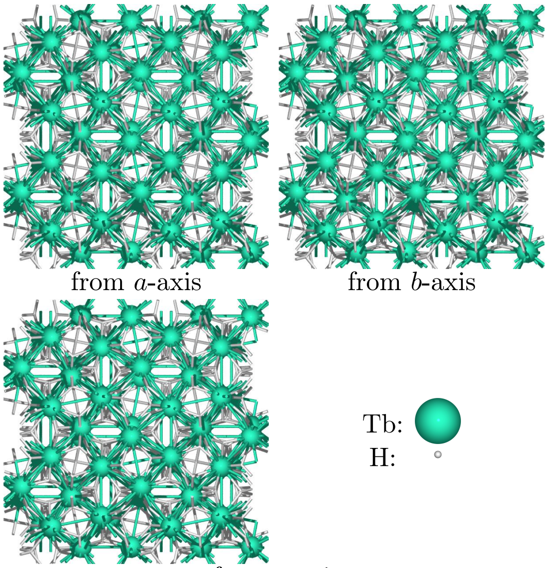

comment:
Accurate calculation.

Primitive structure:

a: 7.5190 Å, b: 7.5190 Å, c: 7.5190 Å
α: 109.47°, β: 109.47°, γ: 109.47°

Tb	[0.9178, 0.9178, 0.9178]
Tb	[0.0822, 0.5000, 1.0000]
Tb	[0.5000, 1.0000, 0.0822]
Tb	[1.0000, 0.0822, 0.5000]
Tb	[0.5000, 1.0000, 0.5822]
Tb	[1.0000, 0.5822, 0.5000]
Tb	[0.5822, 0.5000, 1.0000]
Tb	[0.4178, 0.4178, 0.4178]
H	[0.3728, 0.6549, 0.9706]
H	[0.8451, 0.2179, 0.3157]
H	[0.5294, 0.1843, 0.4022]
H	[0.1272, 0.0978, 0.2821]
H	[0.0978, 0.2821, 0.1272]
H	[0.2821, 0.1272, 0.0978]
H	[0.8728, 0.4706, 0.1549]
H	[0.8157, 0.7179, 0.3451]
H	[0.2500, 0.6250, 0.3750]
H	[0.3750, 0.2500, 0.6250]
H	[0.1250, 0.7500, 0.8750]
H	[0.8750, 0.1250, 0.7500]
H	[0.6250, 0.3750, 0.2500]
H	[0.7500, 0.8750, 0.1250]
H	[0.6272, 0.7821, 0.5978]
H	[0.2179, 0.3157, 0.8451]
H	[0.9706, 0.3728, 0.6549]
H	[0.6549, 0.9706, 0.3728]
H	[0.4022, 0.5294, 0.1843]
H	[0.1843, 0.4022, 0.5294]
H	[0.3157, 0.8451, 0.2179]
H	[0.0294, 0.9022, 0.6843]
H	[0.3451, 0.8157, 0.7179]
H	[0.4706, 0.1549, 0.8728]
H	[0.9022, 0.6843, 0.0294]
H	[0.7179, 0.3451, 0.8157]
H	[0.1549, 0.8728, 0.4706]
H	[0.5978, 0.6272, 0.7821]
H	[0.7821, 0.5978, 0.6272]
H	[0.6843, 0.0294, 0.9022]

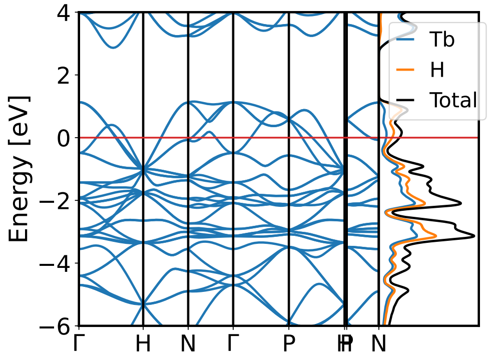

Electron band structure

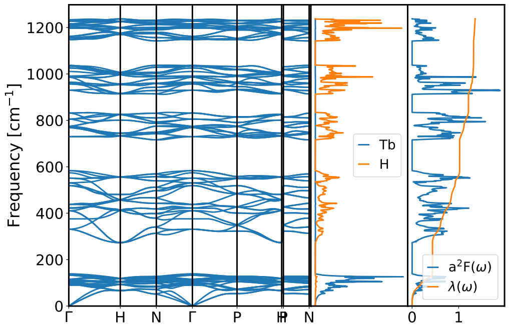

Phonon band structure

#9: Th_4H_{15}

**mat id**	agm003245870
**spg**	220
**nsites**	38
**e above hull**	0.000 eV
**e form**	-0.578 eV
**decomposition**	Th_4H_{15}
**ecutwfc**	136.0 Ry
**kpts coarse**	8×8×8
**kpts fine**	16×16×16
**qpts**	2×2×2
λ	0.381
$\omega_{\mathbf{log}}$	368 K
ω_2	837 K
$T_{\mathbf{c}}^{\mathbf{Mcmillan}}$	1.2 K
$T_{\mathbf{c}}^{\mathbf{Allen\text{-}Dynes}}$	1.2 K
$T_{\mathbf{c}}^{\mathbf{Eliashberg}}$	1.2 K

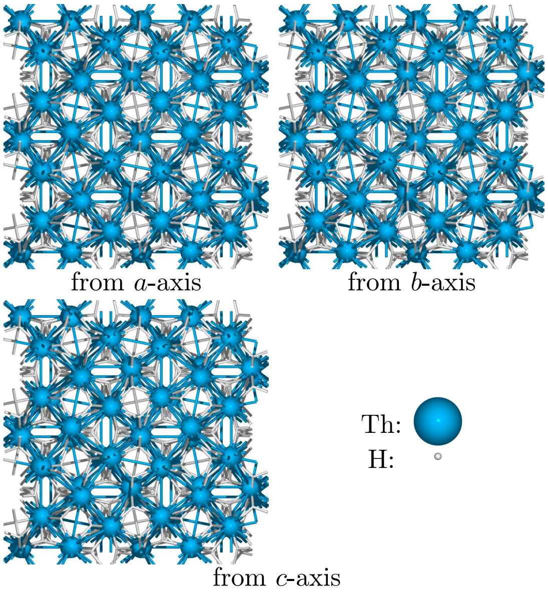

comment:
Accurate calculation.

Primitive structure:

a: 7.8597 Å, b: 7.8597 Å, c: 7.8597 Å
α: 109.47°, β: 109.47°, γ: 109.47°

```
Th  [0.9176, 0.5000, 1.0000]
Th  [0.5000, 1.0000, 0.4176]
Th  [0.0000, 0.4176, 0.5000]
Th  [0.0824, 0.0824, 0.0824]
Th  [0.0000, 0.9176, 0.5000]
Th  [0.5824, 0.5824, 0.5824]
Th  [0.5000, 1.0000, 0.9176]
Th  [0.4176, 0.5000, 0.0000]
 H  [0.6519, 0.1856, 0.2812]
 H  [0.2188, 0.4043, 0.3706]
 H  [0.1856, 0.2812, 0.6519]
 H  [0.0957, 0.3144, 0.9663]
 H  [0.2812, 0.6519, 0.1856]
 H  [0.1294, 0.5337, 0.8481]
 H  [0.4663, 0.8144, 0.5957]
 H  [0.7188, 0.8706, 0.9043]
 H  [0.6250, 0.7500, 0.3750]
 H  [0.2500, 0.1250, 0.8750]
 H  [0.7500, 0.3750, 0.6250]
 H  [0.1250, 0.8750, 0.2500]
 H  [0.3750, 0.6250, 0.7500]
 H  [0.8750, 0.2500, 0.1250]
 H  [0.7812, 0.6856, 0.1519]
 H  [0.3144, 0.9663, 0.0957]
 H  [0.3706, 0.2188, 0.4043]
 H  [0.9663, 0.0957, 0.3144]
 H  [0.5337, 0.8481, 0.1294]
 H  [0.4043, 0.3706, 0.2188]
 H  [0.8481, 0.1294, 0.5337]
 H  [0.9043, 0.7188, 0.8706]
 H  [0.8144, 0.5957, 0.4663]
 H  [0.1519, 0.7812, 0.6856]
 H  [0.6856, 0.1519, 0.7812]
 H  [0.3481, 0.0337, 0.6294]
 H  [0.8706, 0.9043, 0.7188]
 H  [0.6294, 0.3481, 0.0337]
 H  [0.5957, 0.4663, 0.8144]
 H  [0.0337, 0.6294, 0.3481]
```

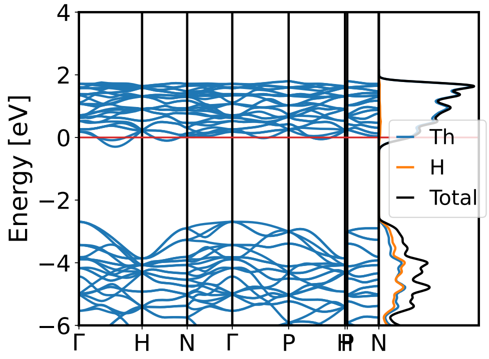

Electron band structure

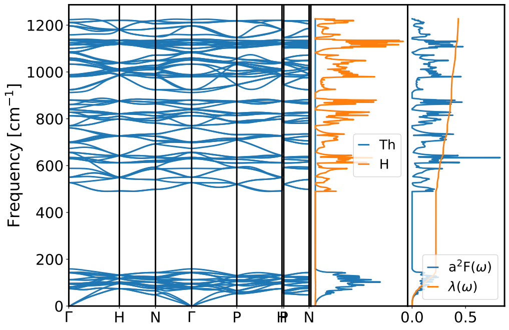

Phonon band structure

#10: Ti_4H_{15}

**mat id**	agm073025883
**spg**	220
**nsites**	38
**e above hull**	0.095 eV
**e form**	-0.274 eV
**decomposition**	TiH_2, H_2
**ecutwfc**	92.0 Ry
**kpts coarse**	8×8×8
**kpts fine**	16×16×16
**qpts**	2×2×2
λ	0.522
ω_{log}	625 K
ω_2	947 K
T_c^{Mcmillan}	9.0 K
$T_c^{\mathrm{Allen\text{-}Dynes}}$	9.2 K
$T_c^{\mathrm{Eliashberg}}$	9.6 K

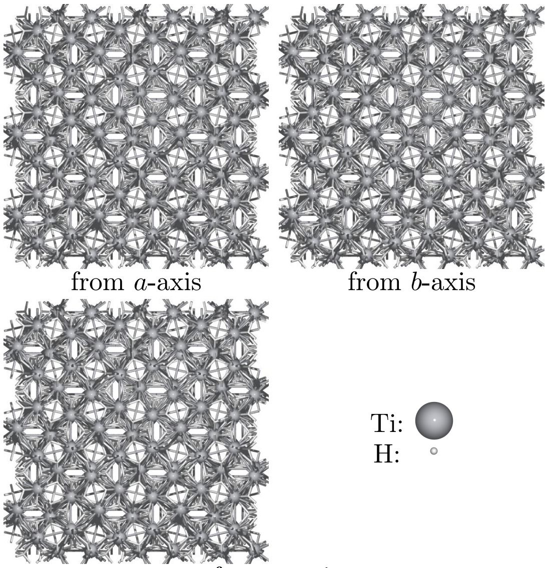

comment:
Accurate calculation.

Primitive structure:

a: 6.4457 Å, b: 6.4457 Å, c: 6.4457 Å
α: 109.47°, β: 109.47°, γ: 109.47°

Ti [0.5958, 0.5000, 0.0000]
Ti [0.9042, 0.9042, 0.9042]
Ti [0.0958, 0.5000, 0.0000]
Ti [0.0000, 0.0958, 0.5000]
Ti [0.0000, 0.5958, 0.5000]
Ti [0.4042, 0.4042, 0.4042]
Ti [0.5000, 0.0000, 0.0958]
Ti [0.5000, 0.0000, 0.5958]
H [0.8890, 0.4680, 0.1555]
H [0.2500, 0.6250, 0.3750]
H [0.5790, 0.6110, 0.7665]
H [0.4680, 0.1555, 0.8890]
H [0.1555, 0.8890, 0.4680]
H [0.5320, 0.1875, 0.4210]
H [0.6110, 0.7665, 0.5790]
H [0.3125, 0.8445, 0.2335]
H [0.8445, 0.2335, 0.3125]
H [0.3445, 0.8125, 0.7335]
H [0.7335, 0.3445, 0.8125]
H [0.3890, 0.6555, 0.9680]
H [0.1250, 0.7500, 0.8750]
H [0.9210, 0.6875, 0.0320]
H [0.3750, 0.2500, 0.6250]
H [0.1875, 0.4210, 0.5320]
H [0.2665, 0.1110, 0.0790]
H [0.6555, 0.9680, 0.3890]
H [0.4210, 0.5320, 0.1875]
H [0.8750, 0.1250, 0.7500]
H [0.7665, 0.5790, 0.6110]
H [0.6875, 0.0320, 0.9210]
H [0.0790, 0.2665, 0.1110]
H [0.0320, 0.9210, 0.6875]
H [0.2335, 0.3125, 0.8445]
H [0.8125, 0.7335, 0.3445]
H [0.1110, 0.0790, 0.2665]
H [0.7500, 0.8750, 0.1250]
H [0.9680, 0.3890, 0.6555]
H [0.6250, 0.3750, 0.2500]

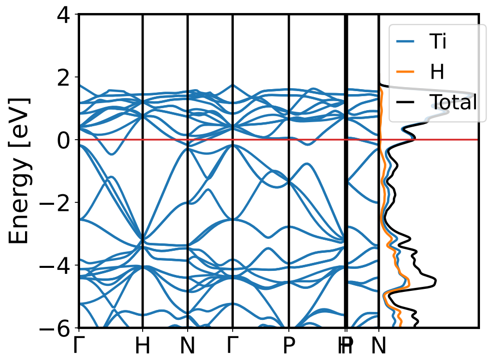

Electron band structure

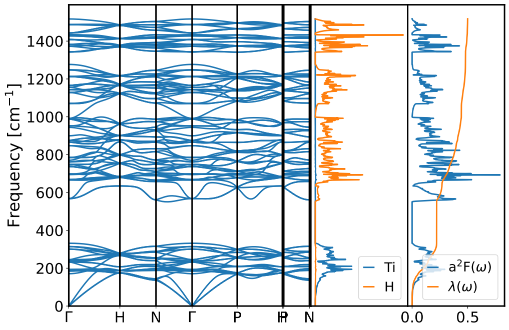

Phonon band structure

#11: Tm_4H_{15}

**mat id**	agm006249799
**spg**	220
**nsites**	38
**e above hull**	0.227 eV
**e form**	-0.375 eV
**decomposition**	TmH_3, H_2
**ecutwfc**	84.0 Ry
**kpts coarse**	8×8×8
**kpts fine**	16×16×16
**qpts**	2×2×2
λ	1.225
$\omega_{\mathbf{log}}$	495 K
ω_2	824 K
$T_{\mathbf{c}}^{\mathbf{Mcmillan}}$	45.5 K
$T_{\mathbf{c}}^{\mathbf{Allen\text{-}Dynes}}$	50.4 K
$T_{\mathbf{c}}^{\mathbf{Eliashberg}}$	56.4 K

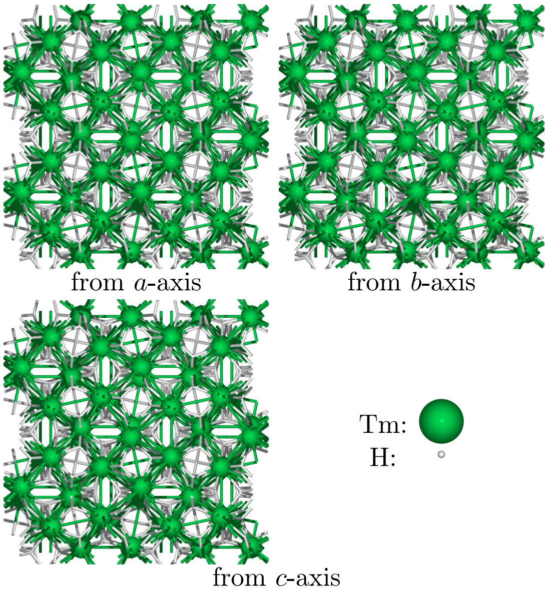

comment:
Accurate calculation.

Primitive structure:

a: 7.3238 Å, b: 7.3238 Å, c: 7.3238 Å
α: 109.47°, β: 109.47°, γ: 109.47°

Tm	[0.9143, 0.9143, 0.9143]
Tm	[0.0857, 0.5000, 0.0000]
Tm	[0.5000, 0.0000, 0.0857]
Tm	[0.0000, 0.0857, 0.5000]
Tm	[0.5000, 0.0000, 0.5857]
Tm	[0.0000, 0.5857, 0.5000]
Tm	[0.5857, 0.5000, 0.0000]
Tm	[0.4143, 0.4143, 0.4143]
H	[0.3778, 0.6563, 0.9723]
H	[0.8437, 0.2214, 0.3160]
H	[0.5277, 0.1840, 0.4054]
H	[0.1222, 0.0946, 0.2786]
H	[0.0946, 0.2786, 0.1222]
H	[0.2786, 0.1222, 0.0946]
H	[0.8778, 0.4723, 0.1563]
H	[0.8160, 0.7214, 0.3437]
H	[0.2500, 0.6250, 0.3750]
H	[0.3750, 0.2500, 0.6250]
H	[0.1250, 0.7500, 0.8750]
H	[0.8750, 0.1250, 0.7500]
H	[0.6250, 0.3750, 0.2500]
H	[0.7500, 0.8750, 0.1250]
H	[0.6222, 0.7786, 0.5946]
H	[0.2214, 0.3160, 0.8437]
H	[0.9723, 0.3778, 0.6563]
H	[0.6563, 0.9723, 0.3778]
H	[0.4054, 0.5277, 0.1840]
H	[0.1840, 0.4054, 0.5277]
H	[0.3160, 0.8437, 0.2214]
H	[0.0277, 0.9054, 0.6840]
H	[0.3437, 0.8160, 0.7214]
H	[0.4723, 0.1563, 0.8778]
H	[0.9054, 0.6840, 0.0277]
H	[0.7214, 0.3437, 0.8160]
H	[0.1563, 0.8778, 0.4723]
H	[0.5946, 0.6222, 0.7786]
H	[0.7786, 0.5946, 0.6222]
H	[0.6840, 0.0277, 0.9054]

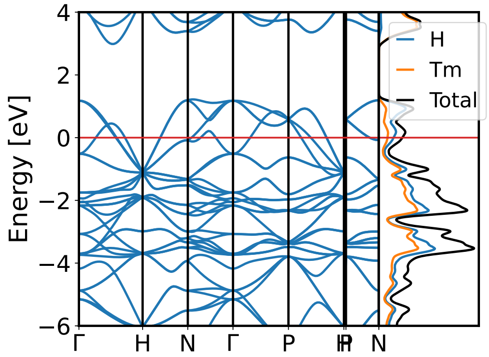

Electron band structure

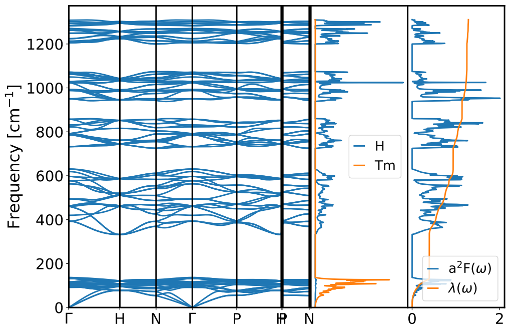

Phonon band structure

#12: Y_4H_{15}

**mat id**	agm006249775
**spg**	220
**nsites**	38
**e above hull**	0.233 eV
**e form**	-0.365 eV
**decomposition**	YH_3, H_2
**ecutwfc**	84.0 Ry
**kpts coarse**	8×8×8
**kpts fine**	16×16×16
**qpts**	2×2×2
λ	1.319
$\omega_{\log}$	474 K
ω_2	751 K
T_c^{Mcmillan}	47.4 K
$T_c^{\mathrm{Allen\text{-}Dynes}}$	53.1 K
$T_c^{\mathrm{Eliashberg}}$	57.4 K

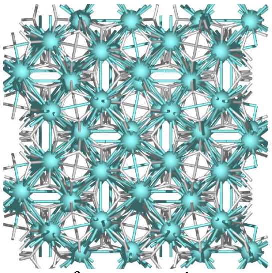

from a-axis

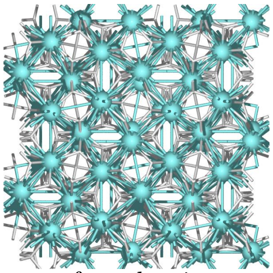

from b-axis

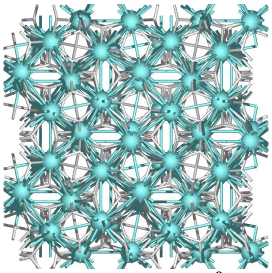

from c-axis

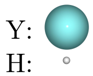

comment:
Accurate calculation.

Primitive structure:

a: 7.4833 Å, b: 7.4833 Å, c: 7.4833 Å
α: 109.47°, β: 109.47°, γ: 109.47°

Y [0.9163, 0.9163, 0.9163]
Y [0.0837, 0.5000, 1.0000]
Y [0.5000, 0.0000, 0.0837]
Y [0.0000, 0.0837, 0.5000]
Y [0.5000, 0.0000, 0.5837]
Y [1.0000, 0.5837, 0.5000]
Y [0.5837, 0.5000, 0.0000]
Y [0.4163, 0.4163, 0.4163]
H [0.3753, 0.6557, 0.9716]
H [0.8443, 0.2195, 0.3158]
H [0.5284, 0.1842, 0.4037]
H [0.1247, 0.0963, 0.2805]
H [0.0963, 0.2805, 0.1247]
H [0.2805, 0.1247, 0.0963]
H [0.8753, 0.4716, 0.1557]
H [0.8158, 0.7195, 0.3443]
H [0.2500, 0.6250, 0.3750]
H [0.3750, 0.2500, 0.6250]
H [0.1250, 0.7500, 0.8750]
H [0.8750, 0.1250, 0.7500]
H [0.6250, 0.3750, 0.2500]
H [0.7500, 0.8750, 0.1250]
H [0.6247, 0.7805, 0.5963]
H [0.2195, 0.3158, 0.8443]
H [0.9716, 0.3753, 0.6557]
H [0.6557, 0.9716, 0.3753]
H [0.4037, 0.5284, 0.1842]
H [0.1842, 0.4037, 0.5284]
H [0.3158, 0.8443, 0.2195]
H [0.0284, 0.9037, 0.6842]
H [0.3443, 0.8158, 0.7195]
H [0.4716, 0.1557, 0.8753]
H [0.9037, 0.6842, 0.0284]
H [0.7195, 0.3443, 0.8158]
H [0.1557, 0.8753, 0.4716]
H [0.5963, 0.6247, 0.7805]
H [0.7805, 0.5963, 0.6247]
H [0.6842, 0.0284, 0.9037]

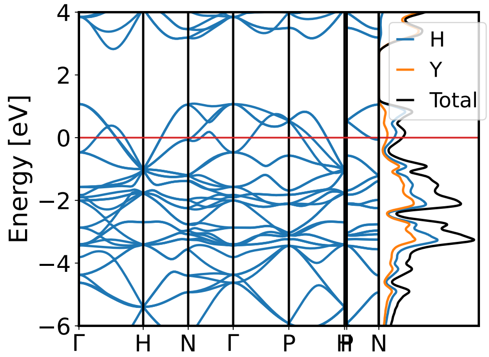

Electron band structure

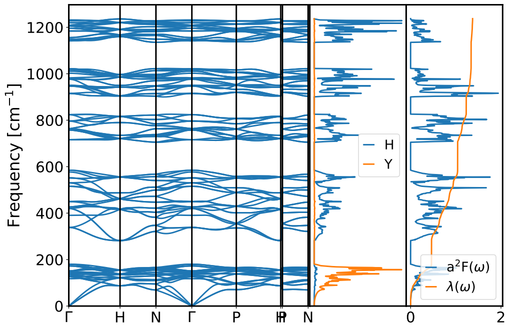

Phonon band structure

#13: Zr_4H_{15}

**mat id**	agm006249778
**spg**	220
**nsites**	38
**e above hull**	0.000 eV
**e form**	-0.429 eV
**decomposition**	Zr_4H_{15}
**ecutwfc**	98.0 Ry
**kpts coarse**	8×8×8
**kpts fine**	16×16×16
**qpts**	2×2×2
λ	0.407
$\omega_{\mathbf{log}}$	607 K
ω_2	1037 K
$T_{\mathbf{c}}^{\mathbf{Mcmillan}}$	2.8 K
$T_{\mathbf{c}}^{\mathbf{Allen\text{-}Dynes}}$	2.9 K
$T_{\mathbf{c}}^{\mathbf{Eliashberg}}$	3.0 K

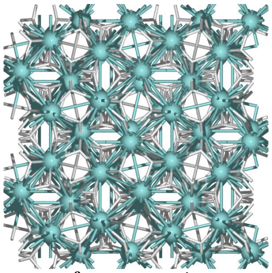

from *a*-axis

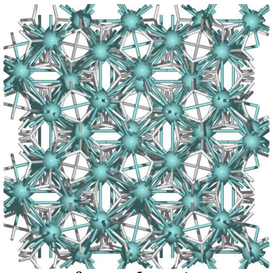

from *b*-axis

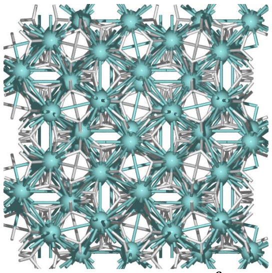

from *c*-axis

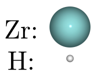

comment:
Accurate calculation.

Primitive structure:

a: 6.9785 Å, *b*: 6.9785 Å, *c*: 6.9785 Å
α: 109.47°, β: 109.47°, γ: 109.47°

Zr [0.9070, 0.9070, 0.9070]
Zr [0.0000, 0.0930, 0.5000]
Zr [0.0930, 0.5000, 1.0000]
Zr [0.5000, 1.0000, 0.0930]
Zr [0.5930, 0.5000, 0.0000]
Zr [0.5000, 1.0000, 0.5930]
Zr [1.0000, 0.5930, 0.5000]
Zr [0.4070, 0.4070, 0.4070]
H [0.9672, 0.3853, 0.6545]
H [0.3127, 0.8455, 0.2308]
H [0.4181, 0.5328, 0.1873]
H [0.2692, 0.1147, 0.0819]
H [0.1147, 0.0819, 0.2692]
H [0.0819, 0.2692, 0.1147]
H [0.1545, 0.8853, 0.4672]
H [0.3455, 0.8127, 0.7308]
H [0.3750, 0.2500, 0.6250]
H [0.6250, 0.3750, 0.2500]
H [0.8750, 0.1250, 0.7500]
H [0.7500, 0.8750, 0.1250]
H [0.2500, 0.6250, 0.3750]
H [0.1250, 0.7500, 0.8750]
H [0.5819, 0.6147, 0.7692]
H [0.8455, 0.2308, 0.3127]
H [0.6545, 0.9672, 0.3853]
H [0.3853, 0.6545, 0.9672]
H [0.1873, 0.4181, 0.5328]
H [0.5328, 0.1873, 0.4181]
H [0.2308, 0.3127, 0.8455]
H [0.6873, 0.0328, 0.9181]
H [0.7308, 0.3455, 0.8127]
H [0.8853, 0.4672, 0.1545]
H [0.0328, 0.9181, 0.6873]
H [0.8127, 0.7308, 0.3455]
H [0.4672, 0.1545, 0.8853]
H [0.7692, 0.5819, 0.6147]
H [0.6147, 0.7692, 0.5819]
H [0.9181, 0.6873, 0.0328]

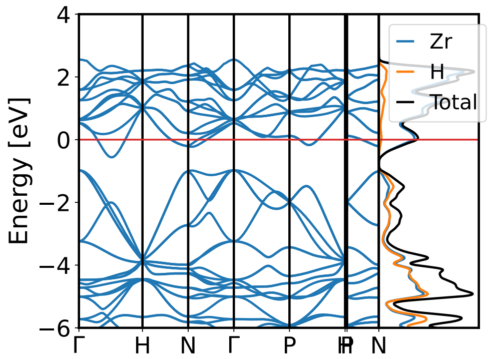

Electron band structure

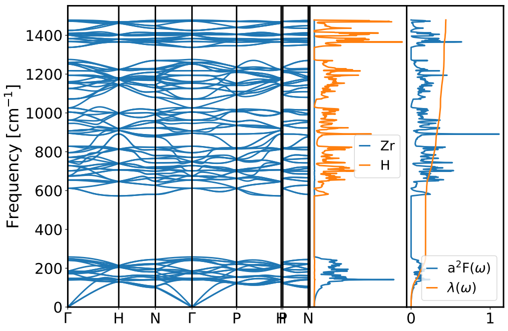

Phonon band structure

#14: $Y(ZrH_5)_3$

**mat id**	agm085201060
**spg**	5
**nsites**	38
**e above hull**	0.005 eV
**e form**	-0.466 eV
**decomposition**	Zr_4H_{15}, YH_3, H_2
**ecutwfc**	98.0 Ry
**kpts coarse**	8×8×8
**kpts fine**	16×16×16
**qpts**	2×2×2
λ	nan
$\omega_{\mathbf{log}}$	nan K
ω_2	nan K
$T_{\mathbf{c}}^{\mathbf{Mcmillan}}$	nan K
$T_{\mathbf{c}}^{\mathbf{Allen\text{-}Dynes}}$	nan K
$T_{\mathbf{c}}^{\mathbf{Eliashberg}}$	nan K

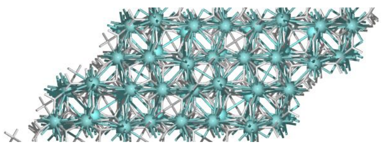

from a-axis

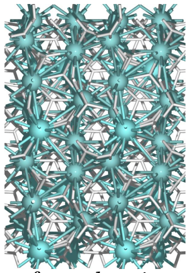

from b-axis

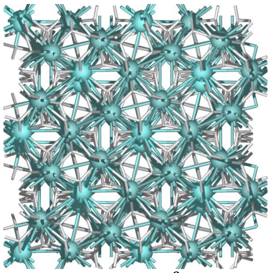

from c-axis

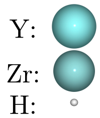

comment:
Accurate calculation. Semiconducting: LO-TO splitting not included in phonons.

Primitive structure:

a: 7.0903 Å, b: 7.0903 Å, c: 8.1754 Å
α: 55.03°, β: 124.97°, γ: 109.21°

Y	[0.0910, 0.9999, 0.3387]
Y	[0.9999, 0.0910, 0.6613]
Zr	[0.8948, 0.4985, 0.7490]
Zr	[0.4985, 0.8948, 0.2510]
Zr	[0.9965, 0.5907, 0.1571]
Zr	[0.4999, 0.3953, 0.2484]
Zr	[0.3953, 0.4999, 0.7516]
Zr	[0.5907, 0.9965, 0.8429]
H	[0.6219, 0.6860, 0.5961]
H	[0.1611, 0.9041, 0.0416]
H	[0.4600, 0.7784, 0.0564]
H	[0.8750, 0.8055, 0.1572]
H	[0.9041, 0.1611, 0.9584]
H	[0.7143, 0.0229, 0.1275]
H	[0.1281, 0.3220, 0.7928]
H	[0.1907, 0.3974, 0.5195]
H	[0.7418, 0.2648, 0.6139]
H	[0.6286, 0.6286, 0.0000]
H	[0.8696, 0.8696, 0.5000]
H	[0.1314, 0.3860, 0.1144]
H	[0.3860, 0.1314, 0.8856]
H	[0.2648, 0.7418, 0.3861]
H	[0.3974, 0.1907, 0.4805]
H	[0.7784, 0.4600, 0.9436]
H	[0.0229, 0.7143, 0.8725]
H	[0.3445, 0.5882, 0.2799]
H	[0.5882, 0.3445, 0.7201]
H	[0.8055, 0.8750, 0.8428]
H	[0.6860, 0.6219, 0.4039]
H	[0.9722, 0.2468, 0.3271]
H	[0.6410, 0.0929, 0.4237]
H	[0.5396, 0.2794, 0.0960]
H	[0.0929, 0.6410, 0.5763]
H	[0.2794, 0.5396, 0.9040]
H	[0.8364, 0.4306, 0.3522]
H	[0.4306, 0.8364, 0.6478]
H	[0.2468, 0.9722, 0.6729]
H	[0.3220, 0.1281, 0.2072]

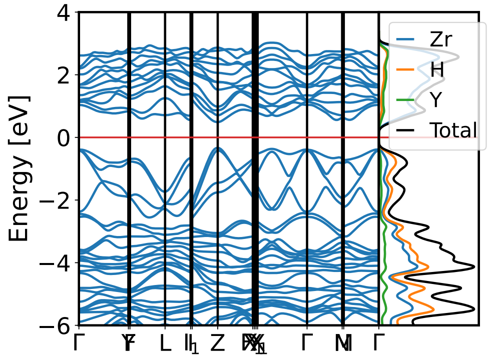

Electron band structure

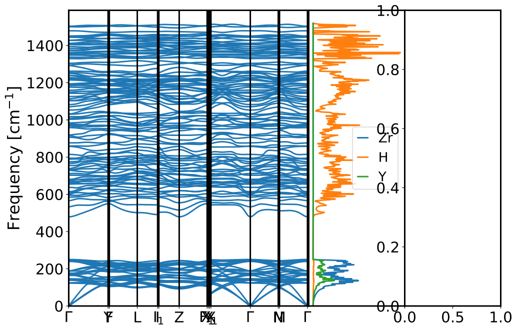

Phonon band structure

#15: $Y_2Zr_2H_{15}$

**mat id**	agm085198323
**spg**	82
**nsites**	38
**e above hull**	0.059 eV
**e form**	-0.455 eV
**decomposition**	Zr_4H_{15}, YH_3, H_2
**ecutwfc**	98.0 Ry
**kpts coarse**	8×8×8
**kpts fine**	16×16×16
**qpts**	2×2×2
λ	0.800
ω_{log}	634 K
ω_2	993 K
$T_{\mathbf{c}}^{\mathbf{Mcmillan}}$	29.7 K
$T_{\mathbf{c}}^{\mathbf{Allen\text{-}Dynes}}$	31.3 K
$T_{\mathbf{c}}^{\mathbf{Eliashberg}}$	31.6 K

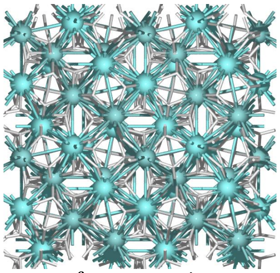

from a-axis

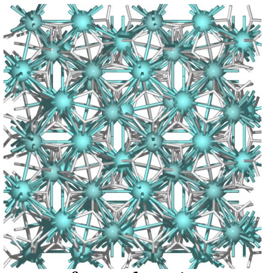

from b-axis

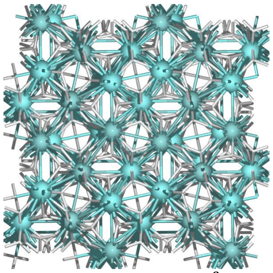

from c-axis

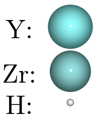

comment:
Accurate calculation.

Primitive structure:

a: 7.2037 Å, b: 7.2037 Å, c: 7.2037 Å
α: 109.63°, β: 109.63°, γ: 109.15°

Y	[0.8717, 0.6237, 0.3367]
Y	[0.4650, 0.1283, 0.7521]
Y	[0.3763, 0.7129, 0.2479]
Y	[0.2871, 0.5350, 0.6633]
Zr	[0.7847, 0.0323, 0.1572]
Zr	[0.3726, 0.2153, 0.2477]
Zr	[0.9677, 0.1249, 0.7523]
Zr	[0.8751, 0.6274, 0.8428]
H	[0.6188, 0.9497, 0.6037]
H	[0.5223, 0.7253, 0.0377]
H	[0.2087, 0.8526, 0.0483]
H	[0.9130, 0.8218, 0.1553]
H	[0.6876, 0.4846, 0.9623]
H	[0.0190, 0.5943, 0.1114]
H	[0.2747, 0.3124, 0.7970]
H	[0.9528, 0.2290, 0.5203]
H	[0.5070, 0.3758, 0.6167]
H	[0.0000, 0.0000, 0.0000]
H	[0.2500, 0.7500, 0.5000]
H	[0.6242, 0.2409, 0.1312]
H	[0.1097, 0.4930, 0.8688]
H	[0.7591, 0.8903, 0.3833]
H	[0.7087, 0.4325, 0.4797]
H	[0.8044, 0.1604, 0.9517]
H	[0.4829, 0.9077, 0.8886]
H	[0.5675, 0.0472, 0.2762]
H	[0.7710, 0.2913, 0.7238]
H	[0.6664, 0.7577, 0.8447]
H	[0.3460, 0.0152, 0.3963]
H	[0.9848, 0.3812, 0.3308]
H	[0.4057, 0.5171, 0.4248]
H	[0.1782, 0.3336, 0.0913]
H	[0.0923, 0.9810, 0.5752]
H	[0.2423, 0.0870, 0.9087]
H	[0.1474, 0.1956, 0.3561]
H	[0.8396, 0.7913, 0.6439]
H	[0.0503, 0.6540, 0.6692]
H	[0.5154, 0.4777, 0.2030]

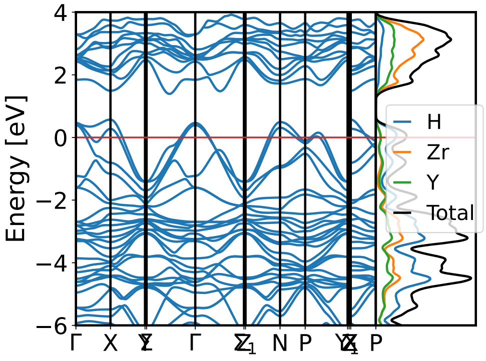

Electron band structure

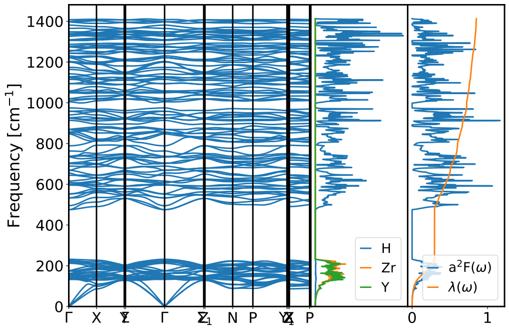

Phonon band structure

#16: $Y_2Zr_2H_{15}$

**mat id**	agm085198331
**spg**	199
**nsites**	38
**e above hull**	0.062 eV
**e form**	-0.452 eV
**decomposition**	Zr_4H_{15}, YH_3, H_2
**ecutwfc**	98.0 Ry
**kpts coarse**	8×8×8
**kpts fine**	16×16×16
**qpts**	2×2×2
λ	0.798
$\omega_{\mathbf{log}}$	649 K
ω_2	1001 K
$T_{\mathbf{c}}^{\mathbf{Mcmillan}}$	30.2 K
$T_{\mathbf{c}}^{\mathbf{Allen\text{-}Dynes}}$	31.8 K
$T_{\mathbf{c}}^{\mathbf{Eliashberg}}$	32.2 K

comment:
Accurate calculation.

Primitive structure:

a: 7.1976 Å, b: 7.1976 Å, c: 7.1976 Å
α: 109.47°, β: 109.47°, γ: 109.47°

Y	[0.5000, 1.0000, 0.5892]
Y	[0.0000, 0.5892, 0.5000]
Y	[0.9108, 0.9108, 0.9108]
Y	[0.5892, 0.5000, 0.0000]
Zr	[0.0893, 0.5000, 0.0000]
Zr	[0.0000, 0.0893, 0.5000]
Zr	[0.4107, 0.4107, 0.4107]
Zr	[0.5000, 0.0000, 0.0893]
H	[0.2106, 0.3068, 0.8386]
H	[0.1280, 0.0962, 0.2894]
H	[0.8386, 0.2106, 0.3068]
H	[0.5318, 0.1932, 0.4038]
H	[0.3068, 0.8386, 0.2106]
H	[0.6614, 0.9682, 0.3720]
H	[0.9295, 0.6842, 0.0389]
H	[0.5705, 0.6094, 0.7548]
H	[0.1306, 0.7500, 0.8806]
H	[0.6194, 0.3694, 0.2500]
H	[0.8806, 0.1306, 0.7500]
H	[0.2500, 0.6194, 0.3694]
H	[0.7500, 0.8806, 0.1306]
H	[0.3694, 0.2500, 0.6194]
H	[0.3547, 0.8158, 0.7452]
H	[0.4038, 0.5318, 0.1932]
H	[0.0962, 0.2894, 0.1280]
H	[0.1932, 0.4038, 0.5318]
H	[0.3720, 0.6614, 0.9682]
H	[0.2894, 0.1280, 0.0962]
H	[0.9682, 0.3720, 0.6614]
H	[0.6094, 0.7548, 0.5705]
H	[0.0389, 0.9295, 0.6842]
H	[0.8158, 0.7452, 0.3547]
H	[0.7452, 0.3547, 0.8158]
H	[0.8906, 0.4611, 0.1453]
H	[0.7548, 0.5705, 0.6094]
H	[0.4611, 0.1453, 0.8906]
H	[0.6842, 0.0389, 0.9295]
H	[0.1453, 0.8906, 0.4611]

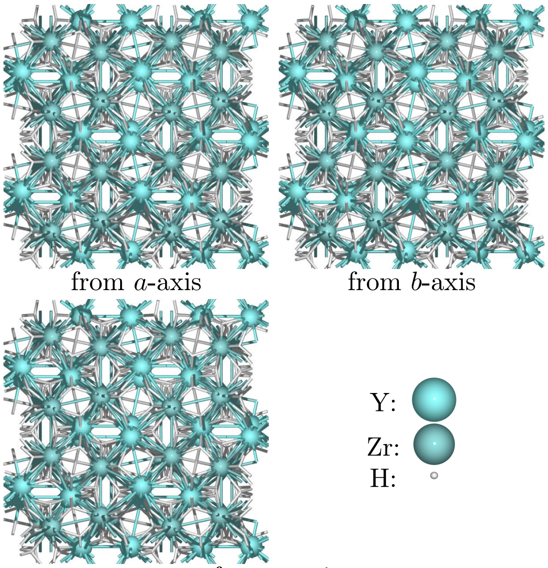

from c-axis

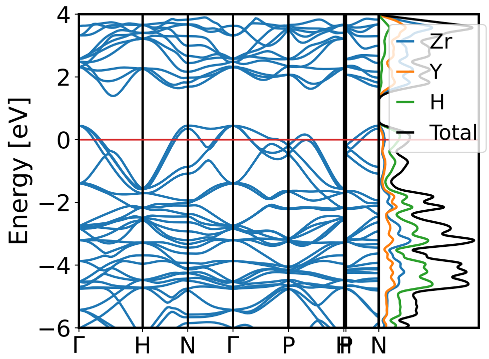

Electron band structure

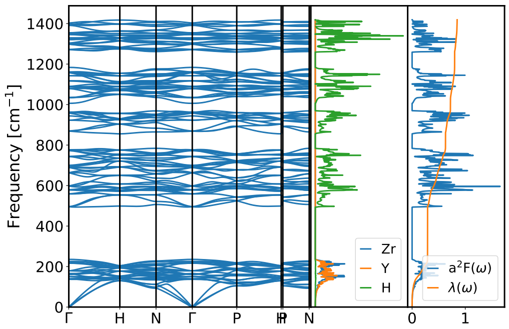

Phonon band structure

#17: $Y_2Zr_2H_{15}$

mat id agm085198344
spg 146
nsites 38
e above hull 0.059 eV
e form -0.454 eV
decomposition Zr_4H_{15}, YH_3, H_2
ecutwfc 98.0 Ry
kpts coarse 8×8×8
kpts fine 16×16×16
qpts 2×2×2
λ 0.794
ω_{log} 636 K
ω_2 996 K
$T_{\mathrm{c}}^{\mathrm{Mcmillan}}$ 29.4 K
$T_{\mathrm{c}}^{\mathrm{Allen\text{-}Dynes}}$ 30.9 K
$T_{\mathrm{c}}^{\mathrm{Eliashberg}}$ 31.2 K

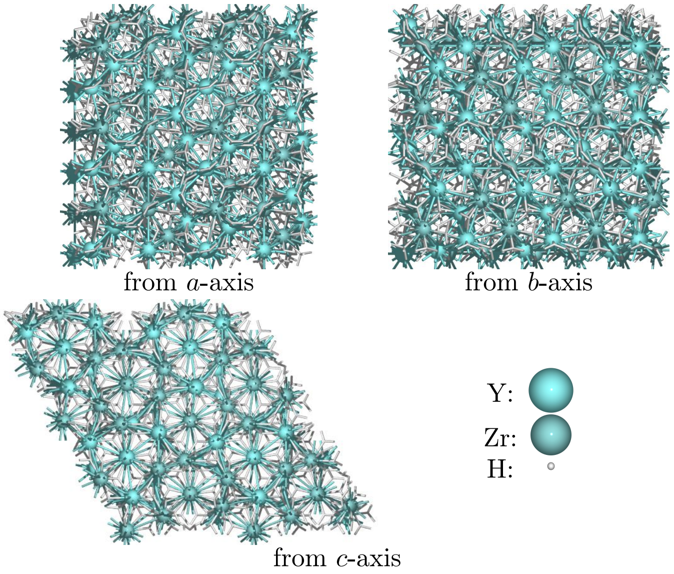

comment:
Accurate calculation.

Primitive structure:

a: 7.2038 Å, b: 7.2038 Å, c: 7.2038 Å
α: 109.47°, β: 109.47°, γ: 109.47°

Y [0.0868, 0.0868, 0.0868]
Y [0.5004, 0.9113, 0.9989]
Y [0.9989, 0.5004, 0.9113]
Y [0.9113, 0.9989, 0.5004]
Zr [0.0031, 0.5051, 0.4123]
Zr [0.4123, 0.0031, 0.5051]
Zr [0.5051, 0.4123, 0.0031]
Zr [0.5926, 0.5926, 0.5926]
H [0.3566, 0.6090, 0.0342]
H [0.7606, 0.1460, 0.6872]
H [0.8029, 0.4695, 0.5701]
H [0.8945, 0.8532, 0.7114]
H [0.7114, 0.8945, 0.8532]
H [0.8532, 0.7114, 0.8945]
H [0.5426, 0.1299, 0.8405]
H [0.2913, 0.1894, 0.6617]
H [0.3657, 0.7378, 0.6143]
H [0.7378, 0.6143, 0.3657]
H [0.2552, 0.8733, 0.1349]
H [0.8733, 0.1349, 0.2552]
H [0.6143, 0.3657, 0.7378]
H [0.1349, 0.2552, 0.8733]
H [0.2263, 0.3926, 0.4212]
H [0.6872, 0.7606, 0.1460]
H [0.6090, 0.0342, 0.3566]
H [0.0342, 0.3566, 0.6090]
H [0.4695, 0.5701, 0.8029]
H [0.5701, 0.8029, 0.4695]
H [0.1460, 0.6872, 0.7606]
H [0.1075, 0.9696, 0.3255]
H [0.1894, 0.6617, 0.2913]
H [0.8405, 0.5426, 0.1299]
H [0.3255, 0.1075, 0.9696]
H [0.6617, 0.2913, 0.1894]
H [0.1299, 0.8405, 0.5426]
H [0.3926, 0.4212, 0.2263]
H [0.4212, 0.2263, 0.3926]
H [0.9696, 0.3255, 0.1075]

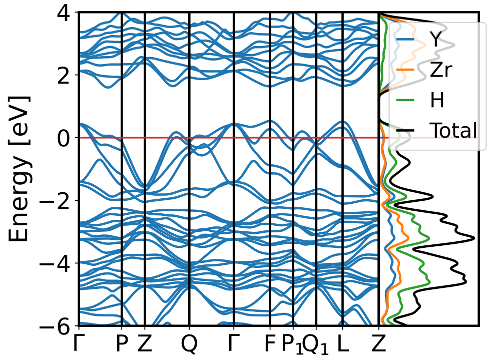

Electron band structure

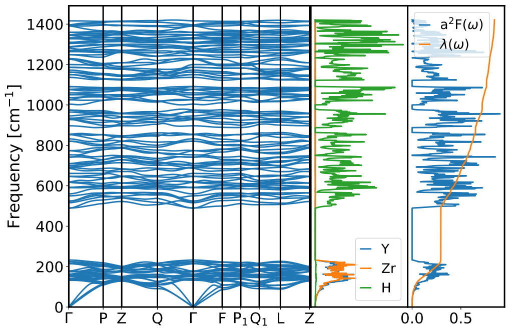

Phonon band structure

#18: Y_3ZrH_{15}

**mat id**	agm121167433
**spg**	5
**nsites**	38
**e above hull**	0.135 eV
**e form**	-0.421 eV
**decomposition**	Zr_4H_{15}, YH_3, H_2
**ecutwfc**	98.0 Ry
**kpts coarse**	8×8×8
**kpts fine**	16×16×16
**qpts**	2×2×2
λ	1.150
ω_{log}	581 K
ω_2	888 K
$T_{\mathbf{c}}^{\mathbf{Mcmillan}}$	49.3 K
$T_{\mathbf{c}}^{\mathbf{Allen\text{-}Dynes}}$	54.0 K
$T_{\mathbf{c}}^{\mathbf{Eliashberg}}$	57.9 K

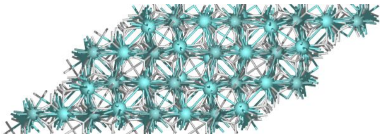

from a-axis

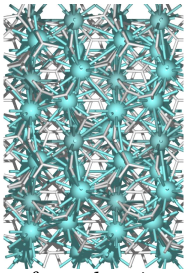

from b-axis

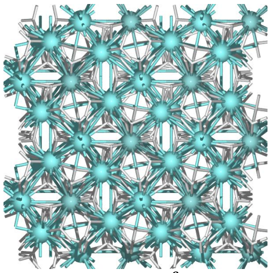

from c-axis

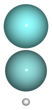

comment:
Accurate calculation.

Primitive structure:

a: 7.3166 Å, b: 7.3166 Å, c: 8.4062 Å
α: 55.20°, β: 124.80°, γ: 109.06°

Y	[0.4989, 0.0821, 0.6658]
Y	[0.0821, 0.4989, 0.3342]
Y	[0.4087, 0.0025, 0.2447]
Y	[0.5839, 0.4987, 0.8304]
Y	[0.4987, 0.5839, 0.1696]
Y	[0.0025, 0.4087, 0.7553]
Zr	[0.9984, 0.9101, 0.7483]
Zr	[0.9101, 0.9984, 0.2517]
H	[0.3088, 0.3714, 0.3417]
H	[0.0737, 0.8545, 0.2185]
H	[0.2226, 0.5188, 0.6304]
H	[0.1800, 0.1130, 0.8916]
H	[0.8545, 0.0737, 0.7815]
H	[0.9484, 0.2588, 0.5571]
H	[0.7077, 0.8780, 0.0444]
H	[0.6421, 0.8230, 0.2883]
H	[0.7677, 0.2230, 0.9019]
H	[0.3707, 0.3707, 0.0000]
H	[0.1129, 0.1129, 0.5000]
H	[0.6442, 0.8826, 0.6155]
H	[0.8826, 0.6442, 0.3845]
H	[0.2230, 0.7677, 0.0981]
H	[0.8230, 0.6421, 0.7117]
H	[0.5188, 0.2226, 0.3696]
H	[0.2588, 0.9484, 0.4429]
H	[0.4100, 0.6549, 0.4583]
H	[0.6549, 0.4100, 0.5417]
H	[0.1130, 0.1800, 0.1084]
H	[0.3714, 0.3088, 0.6583]
H	[0.7913, 0.0319, 0.4109]
H	[0.9104, 0.3254, 0.1346]
H	[0.7201, 0.4661, 0.1575]
H	[0.3254, 0.9104, 0.8654]
H	[0.4661, 0.7201, 0.8425]
H	[0.6059, 0.1428, 0.0684]
H	[0.1428, 0.6059, 0.9316]
H	[0.0319, 0.7913, 0.5891]
H	[0.8780, 0.7077, 0.9556]

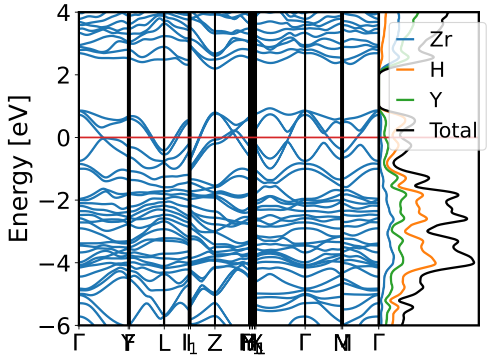

Electron band structure

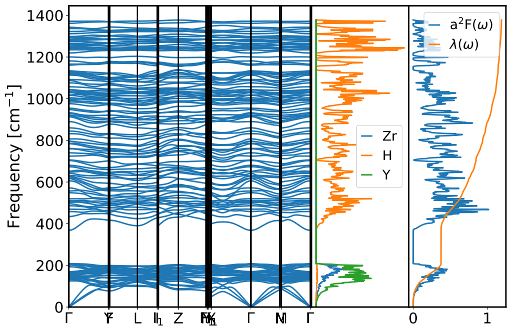

Phonon band structure

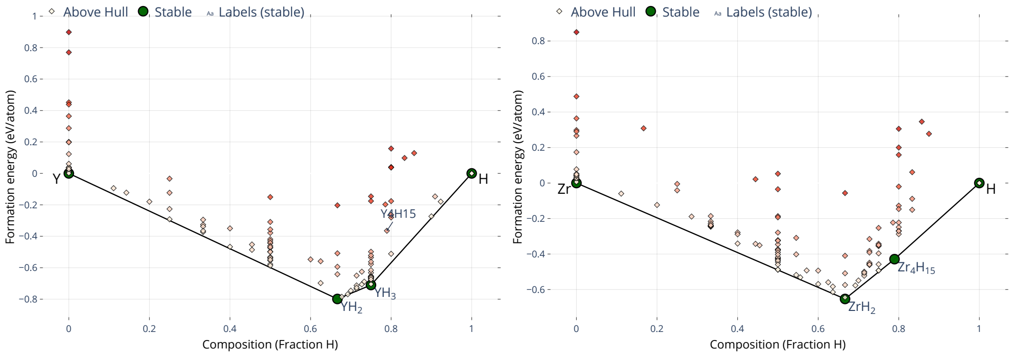

FIG. S1. Binary phase diagram of (left) Y–H and (right) Zr–H systems at 0 GPa.

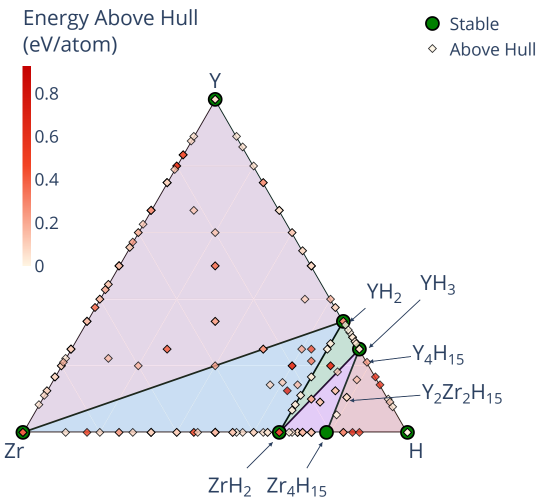

FIG. S2. Ternary phase diagram of Y-Zr–H system at 0 GPa. The phases on and above the hull are depicted as circles and squares, respectively.

TABLE S1. Formation enthalpy (ΔH, in eV/atom) of X_4H_{15} compounds at pressure range from 5-80 GPa. The missing values correspond to pressures where the compounds are dynamically unstable.

**Comp.**	**P**	ΔH	**Comp.**	**P**	ΔH	**Comp.**	**P**	ΔH	**Comp.**	**P**	ΔH
Dy_4H_{15}	5	-0.482	Ho_4H_{15}	5	-0.492	Er_4H_{15}	5	-0.495	Hf_4H_{15}	5	-0.520
	10	-0.548		10	-0.559		10	-0.564		10	-0.590
	20	-		20	-		20	-		20	-
	40	-0.770		40	-0.782		40	-0.792		40	-0.818
	80	-0.877		80	-0.913		80	-0.922		80	-0.939
Tb_4H_{15}	5	-0.474	Nb_4H_{15}	5	-0.204	Th_4H_{15}	5	-0.596	Ti_4H_{15}	5	-0.412
	10	-		10	-0.269		10	-0.630		10	-0.484
	20	-		20	-0.356		20	-		20	-0.586
	40	-0.755		40	-0.459		40	-		40	-0.722
	80	-0.846		80	-0.561		80	-0.704		80	-0.891
Tm_4H_{15}	5	-0.500	Y_4H_{15}	5	-0.484	Zr_4H_{15}	5	-0.560	Lu_4H_{15}	5	-0.505
	10	-0.570		10	-		10	-0.629		10	-0.578
	20	-		20	-		20	-0.703		20	-0.677
	40	-0.799		40	-0.804		40	-0.810		40	-
	80	-0.942		80	-0.936		80	-0.910		80	-0.964

TABLE S2. Superconducting properties of the $Y_2Zr_2H_{15}$ and Y_3ZrH_{15} compounds under pressure.

Compound	Pressure (GPa)	T_c^{AD} (K)	λ	$\omega_{\log}$ (K)
$Y_2Zr_2H_{15}$	-10	39.7	1.899	291
	-5	40.2	1.047	550
	10	24.8	0.699	714
	20	20.3	0.634	750
	40	20.0	0.625	771
	80	36.7	0.842	709
Y_3ZrH_{15}	-5	54.5	1.504	479
	10	45.4	1.058	600
	20	48.2	1.128	583
